# Emerging strategies in targeting tumor-resident myeloid cells for cancer immunotherapy

**DOI:** 10.1186/s13045-022-01335-y

**Published:** 2022-08-28

**Authors:** Yi Wang, Kai Conrad Cecil Johnson, Margaret E. Gatti-Mays, Zihai Li

**Affiliations:** 1 Division of Medical Oncology, Pelotonia Institute for Immuno-Oncology, The Ohio State University Comprehensive Cancer Center, Columbus, OH USA; 2 Stefanie Spielman Comprehensive Breast Center, Columbus, OH USA

**Keywords:** Myeloid-derived suppressor cells (MDSCs), Tumor-associated macrophages (TAMs), Dendritic cells, Reprogramming, Recruitment, Proliferation, Polarization, CAR-M, Tumor microenvironment, Myeloid

## Abstract

Immune checkpoint inhibitors targeting programmed cell death protein 1, programmed death-ligand 1, and cytotoxic T-lymphocyte-associated protein 4 provide deep and durable treatment responses which have revolutionized oncology. However, despite over 40% of cancer patients being eligible to receive immunotherapy, only 12% of patients gain benefit. A key to understanding what differentiates treatment response from non-response is better defining the role of the innate immune system in anti-tumor immunity and immune tolerance. Teleologically, myeloid cells, including macrophages, dendritic cells, monocytes, and neutrophils, initiate a response to invading pathogens and tissue repair after pathogen clearance is successfully accomplished. However, in the tumor microenvironment (TME), these innate cells are hijacked by the tumor cells and are imprinted to furthering tumor propagation and dissemination. Major advancements have been made in the field, especially related to the heterogeneity of myeloid cells and their function in the TME at the single cell level, a topic that has been highlighted by several recent international meetings including the 2021 China Cancer Immunotherapy workshop in Beijing. Here, we provide an up-to-date summary of the mechanisms by which major myeloid cells in the TME facilitate immunosuppression, enable tumor growth, foster tumor plasticity, and confer therapeutic resistance. We discuss ongoing strategies targeting the myeloid compartment in the preclinical and clinical settings which include: (1) altering myeloid cell composition within the TME; (2) functional blockade of immune-suppressive myeloid cells; (3) reprogramming myeloid cells to acquire pro-inflammatory properties; (4) modulating myeloid cells via cytokines; (5) myeloid cell therapies; and (6) emerging targets such as Siglec-15, TREM2, MARCO, LILRB2, and CLEVER-1. There is a significant promise that myeloid cell-based immunotherapy will help advance immuno-oncology in years to come.

## Introduction

**Fig. 1:**
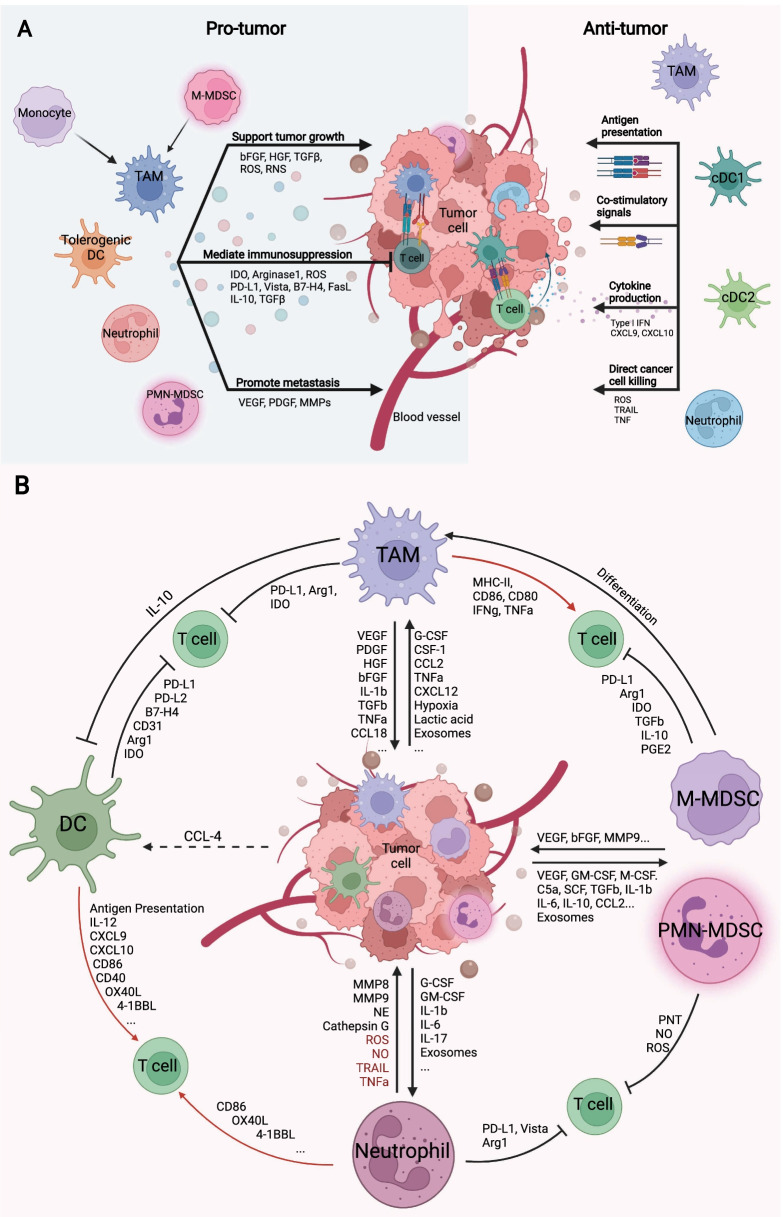
Myeloid cells in the TME: friend or foe? **A**. Myeloid cells can be molded by the TME or therapeutic strategies to exert either pro-tumor or anti-tumor functions. TAMs, tolerogenic DCs, neutrophils, and MDSCs mainly foster cancer progression through supporting tumor cell transition and proliferation, promoting metastasis through enhanced vascularization and preparation of metastatic niche, as well as mediating immunosuppression, through the secretion of soluble factor, extracellular vesicles or direct ligand–receptor interaction. TAMs, DCs, and neutrophils can be programmed toward an anti-tumor phenotype. cDC1s and cDC2s are major APCs that present tumor-associated antigens to T cells and prime T cell responses. TAMs can be reprogrammed to serve as APCs. Both TAMs and DCs, once properly activated, express cytokines such as type I IFN, CXCL9, and CXCL10 to recruit T cells into the TME. Neutrophils can perform direct cancer killing through the generation of ROS or indirect killing induced by death signals such as TRAIL and TNF. **B**. Summary of the cross talk between myeloid cells and tumor cells in the TME. Tumor cells secreted a variety of soluble factors including chemo attractants that recruit myeloid cells. The recruited myeloid cells further amplify these signals and in turn fuel tumor growth and metastasis by producing factors that remodulate surrounding tissue structure, growth factors, and immunosuppressive molecules. Red: anti-tumor effects; Black: pro-tumor effects

Tumors are often described as “wounds that do not heal” [1]. This is likely due in part to the inhibition of myeloid cells within the tumor microenvironment (TME). Myeloid cells are innate immune cells that function as the front line in host defense against pathogens and play important roles in tissue repair after clearance of pathogens [2]. Myeloid cells are important in all stages of tumor development and orchestrate innate and adaptive immune responses [3–5]. In early stages of tumorigenesis, innate immune cells, including macrophages and dendritic cells (DCs), trigger an inflammatory response to induce myelopoiesis and recruitment of other immune cells to eliminate tumor cells [3, 6–9]. However, failure of cytotoxic immune cells to clear the tumor cells due to somatic mutations results in unresolved, persistent inflammation, which continuously recruits immune cell infiltration and gradually reprograms them to support tumorigenesis [10–13]. Myeloid cells, including macrophages, DCs, neutrophils, monocytes, and myeloid-derived suppressor cells (MDSCs) imprinted by the TME, display distinct yet overlapping functions (Fig. 1). Given the development of multi-omics technologies, myeloid cells are now known to have high heterogeneity and complexity, which both create challenges and have implications for the development of myeloid cell-targeting immunotherapies [11, 14–18].

Targeting myeloid cells with immunotherapy was discussed at length during the 2021 China Cancer Immunotherapy workshop in Beijing, the sixth annual conference organized by the Chinese American Hematologist and Oncologist Network (CAHON), China Center for Food and Drug International Exchange (CCFDIE), China National Medical Product Administration (NMPA), and Tsinghua University. Researchers from both the USA and China discussed their most recent work on myeloid cells in immuno-oncology, ranging from the deconvolution of the myeloid compartment in the TME to discovering novel targets for manipulating myeloid cells for improved immunotherapy. In this review, we discuss the mechanisms of myeloid cell-mediated tumor immunity and evasion. We will highlight selected approaches for modulation of myeloid cells and include data presented at the 2021 China Cancer Immunotherapy Workshop. Finally, we provide a comprehensive review of the ongoing clinical trials involving novel agents that target myeloid cells for the purpose of cancer treatment.

## Major myeloid cell populations in the tumor microenvironment and their mechanism of immunosuppression

### Tumor-associated macrophages

Tumor-associated macrophages (TAMs) are the most abundant immune cells within the TME. High infiltration of TAMs or enrichment of the TAM gene signature is associated with poor prognosis in solid tumors including breast cancer, bladder cancer, and cervical cancer [11, 19–21]. Macrophages have different origins dependent on tissue types and thus are not always categorically “myeloid” cells. They can be yolk sac- or fetal liver-derived tissue-resident macrophages (TRMs) in addition to those differentiated from hematopoietic stem cells (HSCs) [22–24]. TAMs include both TRM and monocyte-derived macrophages [17, 25–29]. Despite differential origins, TAMs are programmed by the surrounding environment to primarily suppress anti-tumor immunity, while the anti-tumor functions of TAMs in response to certain treatments, such as low-dose irradiation and histone deacetylase (HDAC) inhibitors, have also been described [30, 31].

Macrophages can be polarized in vitro into pro-inflammatory M1 phenotype by IFNγ and lipopolysaccharide (LPS) treatment, or anti-inflammatory M2 phenotype when treated with interleukin-4 (IL-4) [32–34]. M1 macrophages produce pro-inflammatory cytokines such as tumor necrosis factor α (TNFα), IL-1β, IL-12, and IL-18 and upregulate major histocompatibility complex (MHC) class II (MHC-II) as well as co-stimulatory molecules including CD80 and CD86. M2 macrophages are characterized as immunosuppressive through the expression of immune inhibitory molecules including transforming growth factor β (TGFβ), IL-10, arginase 1 (Arg1), and CD206. The M1/M2 macrophages retain plasticity after polarization, which can be reversed depending on the culture condition [35]. However, the M1/M2 classification is oversimplified for TAMs because TAMs consist of a heterogenous population and express both M1 and M2 signatures phenotypically [36].

The TME is a hostile environment due to rapid tumor growth and high metabolic demand and is characterized by hypoxia, restricted nutrition availability, acidosis, and other factors. The interplay between a tumor cell and the surrounding cells pressures the infiltrating immune cells to shift their phenotypes to adapt to the TME [37]. Macrophages are initially recruited to the tumor sites through macrophage colony-stimulating factor 1 (CSF-1) signaling [38], and amplified through a variety of cytokines including C–C motif chemokine ligand 2 (CCL2), TNFα, vascular endothelial growth factor (VEGF), C-X-C motif chemokine ligand 12 (CXCL12), and TGFβ [26, 39–42]. Hypoxia and lactic acid polarized TAMs produce a wide range of soluble factors including VEGF, platelet-derived growth factor (PDGF), hepatocyte growth factor (HGF), and basic fibroblast growth factors (bFGF) as well as inflammatory cytokines including TGFβ, IL-1β, TNFα, and CCL18 to promote tumor growth, angiogenesis, tumor plasticity, and metastasis [3, 43, 44].

In addition to soluble factors, tumor cells may also skew myeloid cell differentiation and phenotype through extracellular vesicles (EV) which transfer DNA, RNA, proteins, lipids, metabolites, or miRNAs [45, 46]. Tumor-derived exosomes (TDEs) are a subclass of EV which are taken up by macrophages, induce PD-L1 expression on macrophages and enhance their immunosuppressive capacity [47]. Although the underlying mechanism is not well elucidated, a recent study suggests that TDEs metabolically reprogram macrophages by engaging with toll-like receptors 2 (TLR2) and triggering MyD88 and NF-κB signaling, leading to increased glycolytic activity, elevated lactate production and polarizing macrophages into an immunosuppressive phenotype [47]. TDEs also engage with other TLRs such as TLR4 and TLR7 on other phagocytes including monocytes and neutrophils in which miRNA, noncoding RNA, and high-mobility group box 1 (HMGB1) transferred by TDEs are implicated in driving the pro-tumor phenotype of these myeloid cells [48–50].

TAMs play a central role in mediating immunosuppression, inhibiting tumor cell clearance by cytotoxic T cells via direct contact or secretion of soluble factors. PD-L1 expression is upregulated in TAMs in mouse models and in human cancers including hepatocellular carcinoma (HCC), melanoma, breast, and ovarian cancer [51–53]. PD-L1 expression on TAMs or other myeloid cells contributes to CD8^+^ T cell suppression and resistance to immune checkpoint inhibitor (ICI) therapy [52, 53]. Another co-inhibitory molecule B7-H4 expressed on TAMs mediates T cell dysfunction in HCC and ovarian cancer [54, 55]. L-Arginine is essential for T cell metabolic fitness and survival as well as the generation of memory T cells [56]. Arginase produced by TAMs depletes L-Arginine in the TME and represses T cell receptor (TCR) expression on activated T cells, resulting in impaired anti-tumor T cell responses [57]. TAMs also act through intermediate cells to suppress T cell activity. For example, IL-10 secreted by TAMs promotes regulatory T cell (Treg) function and inhibits IL-12 production by CD103^+^ DCs, leading to T cell suppression and diminished T cell activation [58]. TAMs confer therapeutic resistance to chemotherapy, immunotherapy, and radiation [59]. In triple-negative breast cancer (TNBC), chemotherapy-induced reactive oxygen species (ROS) which upregulates PD-L1 on TAMs, leading to reduced efficacy of paclitaxel [60]. In a recent study, monocyte-derived macrophages in liver metastasis eliminate anti-tumor CD8^+^ T cells through induction of Fas-dependent apoptosis, thus mediating resistance to ICI therapies and may explain the immunosuppressive TME within the liver [61].

Because of the high abundance, durability, and adaptability of macrophages, TAMs reeducated by the TME play pivotal roles in fueling tumor progression. However, the versatility of TAMs also provides opportunities for manipulation for therapeutic purposes. Although TAMs do not fit nicely into M1/M2 classification, a high M1 signature over M2 signature predicts better survival in ovarian cancer, strengthening the rationale of targeting TAMs for cancer treatment [62]. Strategies ranging from TAM depletion, repolarization, metabolic reprogramming, and even engineered macrophages are being developed. Given the heterogeneity of TAM populations, targeting a specific TAM subpopulation may enhance the likelihood of effective tumor suppression. In the 2021 China Cancer Immunotherapy Workshop, Edgar G. Engleman (Stanford University) noted that a C-type lectin receptor Dectin-2 is most highly expressed in TAMs and dictates a highly immunosuppressive phenotype. Accumulation of Dectin-2^+^ TAMs promotes tumor growth in mouse tumor models. However, intratumoral administration of Dectin-2 ligand reprograms TAMs into an immune-activating phenotype and contributes to enhanced anti-tumor immunity. Emerging TAM targets will be discussed later in this review.

### Dendritic cells

Dendritic cells (DCs) are key antigen-presenting cells (APCs) that prime and activate T cells. Despite their low abundance in the TME, DCs play a vital role in bridging innate immunity with adaptive immunity and orchestrating anti-tumor responses by T cells (Fig. 1). Conventional DCs (cDCs) and plasmacytoid DCs (pDCs) are two major types of DCs. cDCs are differentiated from common DC precursors (CDP) and are divided into cDC1s and cDC2s [63]. The cDC1 population, which are recognized as CD103^+^ DCs in mice and CD141^+^ DCs in humans, can cross-present tumor antigens to CD8^+^ T cells through MHC class I (MHC-I) in addition to stimulating Th1 polarization of CD4^+^ T cells [64–66]. The cross-presentation capacity by cDC1s is critical for priming CD8^+^ T-cell-mediated anti-tumor immunity both in situ and in the lymph nodes [66–69]. cDC1 infiltration correlates with improved clinical outcomes to immunotherapy [66–69]. cDC2s are defined as CD11b^+^ DCs in mouse models and CD1c^+^ in humans. They induce CD4^+^ T cell responses through MHC-II presentation and contribute to immune surveillance in the TME [70, 71]. A recent study found a subset of cDC2s expressing interferon-stimulated genes (ISGs) also has antigen cross-presentation ability and fosters CD8^+^ T cell-dependent anti-tumor immunity [72]. In addition, cDC1s are more effective in MHC-II presentation than cDC2s [73, 74]. cDC1s activate CD4^+^ T cells and are licensed by CD40 signaling via CD4^+^ T cells, for optimal CD8^+^ T cell priming [75].

In addition to the antigen-specific signal by MHC molecules, mature DCs express co-stimulatory molecules such as CD86, CD80, CD40, OX40L, GITRL, and 4-1BBL, which are essential for optimal T cell activation and survival [5]. Type I interferon (IFN) plays an important role in host anti-tumor immunity [76–78]. Activation of the cytosolic DNA sensing pathway mediated by cyclic GMP-AMP synthase (cGAS) and Stimulator of interferon genes (STING) in DCs promote DC maturation and type I IFN production, which augments T cell cytotoxicity to eradicate tumors [79–81]. Tumor-resident DCs stimulated by type I IFN produce CXCL9 and CXCL10 which promotes T cells trafficking to the TME [67]. IL-12 produced by cDC1s is required for anti-tumor immunity by T cells and response to anti-PD-1 therapy [82].

DCs are modulated by the TME to drive immune tolerance. Co-inhibitory molecules such as PD-L1, PD-L2, V-domain immunoglobulin suppressor of T cell activation (VISTA), and CD31 are induced on DCs to restrain T cell function [5, 83–85]. Inflammasomes are cytosolic multiprotein complex triggered by pathogen-associated molecular patterns (PAMPs) or danger-associated molecular patterns (DAMPs), which initiate a pyroptotic inflammatory response [86]. A subset of TIM-3^+^ DCs with reduced DNA uptake capacity suppresses anti-tumor immunity through inflammasome activation, which can be reversed by TIM-3 blockade [87–89]. CTLA-4 ligation with CD80 and CD86 induces indoleamine 2,3-dioxygenase 1 (IDO1) by DCs, which converts the essential amino acid tryptophan to kynurenine, inhibiting T cell proliferation and favoring Treg cell differentiation [90].

During the 2021 China Cancer Immunotherapy Workshop, Miriam Merad (Mount Sinai) discussed the identification of a new DC cluster, “mature DCs enriched in immunoregulatory molecules” (mregDCs), which were present in non-small cell lung cancer (NSCLC), HCC, and colorectal cancer (CRC) using single-cell RNA sequencing and cellular indexing of transcriptomes and epitopes by sequencing (CITE-seq) [91]. These mregDCs co-express immune regulatory genes such as *CD274, Pdcd1lg2*, and *CD200* and maturation genes (*CD40, Ccr7*, and *Il12rb*). Both cDC1s and cDC2s can acquire mregDC signature upon sensing or uptake of tumor-associated antigen, partially driven by IL-4 signaling and AXL signaling, whereas IFNγ is required for IL-12 production by mregDCs. IL-4 blockade enhances IL-12 production in mregDCs and promotes T cell effector function [91].

pDCs are differentiated from CDPs or lymphoid progenitors [92]. They are potent type I IFN producers when encountering pathogens but poor antigen presenters [93]. pDCs in the TME have impaired type I IFN production (mediated in part by TGFβ) and increased induction of Treg differentiation, hence supporting tumor growth in breast and ovarian cancer [94–99]. However, the full role of pDCs in the TME is not yet clear.

### Neutrophils

Neutrophils are the most abundant myeloid population that developed from granulocyte–monocyte progenitors (GMPs) in the bone marrow. The high neutrophil-to-lymphocyte ratio (NLR) in the peripheral blood of cancer patients is associated with poor prognosis in many cancers, including NSCLC, CRC, HCC, and prostate cancer [100–106]. A higher baseline NLR is associated with worse survival and decreased clinical response with ICI therapy across many cancer types including advanced melanoma and NSCLC [107–110]. Neutrophilic infiltration is seen in the majority of solid tumors; however, the prognostic relevance remains controversial and inconsistent [21]. For example, in HCC, a high density of tumor-associated neutrophils is associated with poor prognosis while in CRC, mixed conclusions are reported [111–115].

Circulating neutrophils are divided into two populations based on density: high-density neutrophils (HDNs), and low-density neutrophils (LDNs) that are found within the mononuclear cell fraction after density gradient centrifugation of blood, indicating an immature phenotype [116]. LDNs are pleiotropic and can be either immunosuppressive or pro-inflammatory, depending on the disease context [116]. Tumor-associated neutrophils (TANs) are classified into anti-tumor N1 and pro-tumor N2, which mimics the nomenclature of M1/M2 polarized macrophages [116–118]. The diversity of the neutrophile composition within tissues and variations between disease states likely contributes to these inconsistent implications.

Neutrophil recruitment to the tissues is mainly dependent on CXCL8-CXCR1/CXCR2 axis [119]. Cytokines produced by tumor and surrounding cells such as GM-CSF, G-CSF, and IL-6 stimulate granulopoiesis in the bone marrow and recruit neutrophils to the tumor site [3, 117, 120, 121]. IL-1β and G-CSF prolong neutrophil survival in the TME [122]. Other molecules such as IL-17 produced by γδ T cells are also involved in neutrophil recruitment in the TME [123]. Neutrophils promote cancer progression through both unique and shared mechanisms as TAMs. They can modulate the extracellular matrix (ECM) by producing matrix metallopeptidase (MMP) 8 and 9 along with neutrophil elastase (NE), inducing VEGF production to promote metastasis [124, 125]. They also release ROS and reactive nitrogen species (RNS) to induce DNA damage in epithelial cells to facilitate carcinogenesis [118]. Neutrophils express a wide repertoire of cytokines and inhibitory ligands that mediate immunosuppression via crosstalk with other immune cells. For example, Arg1, PD-L1, and VISTA expressed by neutrophils dampen T cell function in the TME [117].

Despite the pro-tumoral activities discussed above, neutrophils also play anti-tumoral roles and prevent metastasis in the TME. Neutrophils can eliminate cancer cells through ROS-dependent killing, which induces lethal Ca^2+^ influx in target cells, dependent on transient receptor potential melastatin 2 (TRPM2) that is highly expressed in cancer cells [126, 127]. Neutrophils can also elicit tumor-killing functions by the expression of NO, TRAIL, and TNF [128, 129]. In addition to direct killing, neutrophils are shown to express immune stimulatory molecules such as CD86, OX40L, and 4-1BBL to enhance T cell function [130].

Neutrophils are known to form neutrophil extracellular traps (NETs) to confine pathogens from dissemination and exert immune modulatory functions. Like neutrophils themselves, NETs possibly play multifaceted roles in tumor immunity. They potentially facilitate tumor progression by the release of NE, cathepsin G, and MMP9, as well as tumor metastasis [131–134]. NETs may also shield tumor cells and protect them from CD8^+^ T cell and NK cell cytotoxicity [135]. By contrast, there is evidence that NETs degrade pro-inflammatory cytokines and reduce inflammation in chronic inflammation [136], which may have implications for the positive role of NETs in tumor. More studies are required to elucidate the role of NETs in the TME.

### Monocytes

Monocytes are classified into three major populations distinguished by differential expression of CD14 and CD16 in human, and in mouse Ly6c and TREML4: classical (CD14^++^CD16^−^ in human and Ly6c^++^TREML4^−^ in mouse), intermediate (CD14^++^CD16^+^ in human and Ly6c^int^ in mouse) and non-classical (CD14^+^CD16^++^ in human and Ly6c^−^TREML4^++^ in mouse) monocytes [4]. Non-classical monocytes, also known as “patrolling” monocytes, play an important role in maintaining vessel integrity by clearing dying endothelial cells and preventing tumor metastasis [137]. Classical monocytes are more abundant than non-classical and are recruited to tissue via CCL2–CCR2 axis. VEGF-A and CSF-1 play redundant roles in monocyte recruitment [42, 138]. Upon encountering tumor-derived signals, monocytes sequentially differentiate into TAMs, promoting cancer progression, metastasis as well as mediating immunosuppression [4]. Genetic ablation of CSF-1 reduced TAMs infiltration and delayed tumor progression in mouse mammary tumor models [138].

TDEs are reported to modulate monocyte function in different directions depending on the source of TDEs [139–141]. TDEs secreted by highly metastatic melanoma recruit pro-tumor monocytes to the pre-metastatic niche, while TDEs from non-metastatic tumors induce the expansion of anti-tumor “patrolling” monocytes and prevent lung metastasis by clearing tumor cells at the pre-metastatic niche [140].

### Myeloid-derived suppressor cells

During myelopoiesis, immature myeloid cells are found in circulation and tumor sites. They are similar to monocytes and neutrophils but exhibit potent immunosuppressive activity, and are termed MDSCs [142, 143]. There are two types of MDSCs: monocytic MDSC (M-MDSC) and granulocytic/polymorphonuclear MDSC (PMN-MDSC). The distinction of MDSCs from neutrophils and monocytes has long been challenging. In the TME, it is likely that monocytic cells undergo sequential differentiation stages, from monocytes to M-MDSCs, and eventually become TAMs [144]. MHC class II is widely used for distinguishing M-MDSCs (CD14^+^CD15^−^HLD-DR^lo/−^ in human) from monocytes (CD14^+^CD15^−^HLD-DR^hi^ in human) while this may not be sufficient [145]. Although TAMs can be phenotypically distinguished from M-MDSCs in mice through increased expression of F4/80, CD115, and IRF8 (CD68 and CD163 in human) and lower expression of Ly6c and S100A9, a specific marker for M-MDSC is needed to better address the difference in monocytic cells [144]. It is even more difficult to identify PMN-MDSCs from neutrophils because they share the same phenotypical markers and they have overlapped functions. Both LDNs and N2 TANs refer to PMN-MDSCs in cancer-related studies. The lack of a uniform nomenclature for granulocytic cells creates confusion in studying their roles, especially between pro-tumor N2 TANs and PMN-MDSCs [144]. Now more markers such as LOX1 which distinguishes PMN-MDSCs from neutrophils in humans have emerged to help better identify these myeloid cell subpopulations [146].

With more in-depth study of MDSCs using transcriptomic and proteomic technologies, there is an updated view that MDSCs are pathologically activated neutrophils and monocytes during persistent myelopoiesis [145]. Tumor-derived factors such as GM-CSF, CSF-1, and G-CSF signal through signal transducer and activator of transcription 3 (STAT3), CCAAT/enhancer-binding protein β (C/EBPβ) and IRF8 to promote myelopoiesis [147, 148]. Downregulation of IRF8 in myeloid progenitors prevents terminal differentiation, therefore leading to the accumulation of immature myeloid cells [147, 149, 150]. The secondary signals from tumor- and tumor stroma-derived factors including HMGB1, TLRs, TGFβ, and endoplasmic reticulum (ER) stress then pathologically activate MDSCs through STAT6, STAT1, and NF-κb signaling pathways [142, 147, 151].

Like TAMs, MDSCs remodel the TME by producing VEGF, bFGF, and MMP9 to facilitate cancer progression and metastasis [147, 152, 153]. MDSCs also exert immunosuppression by suppressing T cell function through direct ligand–receptor engagement, release of soluble inhibitory cytokines and sequestration of amino acids essential for T cells. In most cancer types, PMN-MDSCs are the major population (~ 80%) of MDSCs [147]. STAT3 phosphorylation is increased in MDSCs and results in elevated nicotinamide adenine dinucleotide phosphate (NADPH) level, leading to ROS accumulation [154]. ROS and ROS-mediated peroxynitrite (PNT) accumulation nitrates TCR and block TCR binding with MHC molecules, impairing T cell responsiveness to antigens [155]. Hyperproduction of PNT also inhibits T cell infiltration by nitrating the chemokines that are responsible for attracting T cells [155–158]. M-MDSCs are rapidly converted to TAMs in tumor hypoxia regions to enhance immunosuppression [159, 160]. Both of PMN-MDSCs and M-MDSCs produce Arg1, iNOS, and IDO1 to suppress T cell function [142, 147, 161]. MDSCs also impair other immune cell functions including DCs, B cells, and NK cells but promote Tregs by producing IL-10 and TGFβ [13, 162–164].

**Fig. 2:**
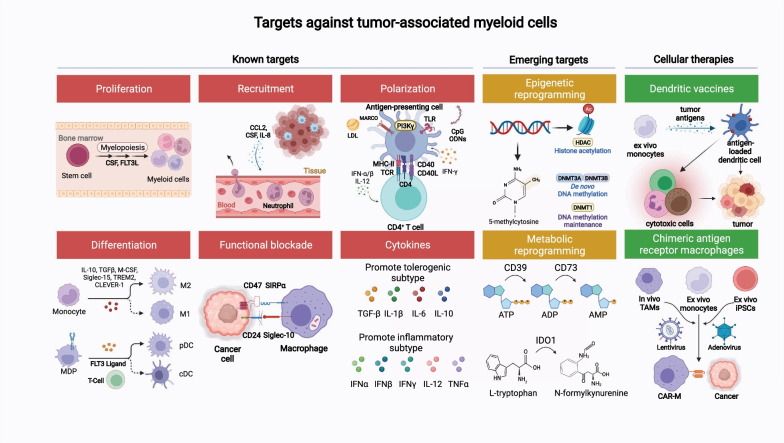
Myeloid-specific targets as immune adjuncts for the management of solid malignancies. Targets were categorized based on their functional role: proliferation, differentiation, recruitment, polarization, functional blockade, cytokine signaling, epigenetic reprogramming, and metabolic reprograming. For cellular therapies, myeloid-specific approaches include dendritic cell vaccines and chimeric antigen receptor macrophages

In summary, the major myeloid cell populations in the TME, including TAMs, DCs, neutrophils, and MDSCs, are “aberrantly programmed” by the TME. Once activated, these cells exert effects on the TME which promote tumor growth. Strategies have been developed to recalibrate these myeloid cells and harness their power to restore anti-tumor immunity (Fig. 2 and Table 1). We will discuss preclinical data and clinical data as it relates to each target. We will also discuss some emerging targets for myeloid cell manipulation.

**Table 1:** Summary of current strategies targeting myeloid cells in clinic

Function	Target molecule [Ref]	Agents	Total # trials		Combination agents	Tumor types	Trial phases
			Monotherapy	Combination			
Proliferation, differentiation, and recruitment	CCR2 [165–172]	BMS-8113160	0	5	PD-1, chemotherapy, radiation, vaccine	CRC, hepatic, lung, pancreatic, renal	1/2, 2
	CSF1R [173–186]	Axatilimab, Emactuzumab, Bezuclastinib, Pexidartinib, Cabiralizumab, Lacnotuzumab, PD-0360324, IMC-CS4	5	14	BRAF, MEK, mTOR, PD-L1, PD-1, TKI, VEGF-A, chemotherapy, radiation, vaccine	Biliary, breast, fallopian, GIST, H&N, mastocytosis, melanoma, myeloid, lymphoma, ovarian, pancreatic, peritoneal, sarcoma, solid	1, 1/2, 2, 3
	CXCR1/2 [187–193]	SX-682, AZD5069, Navarixin	1	7	ADT, CTLA-4, PD-1, PD-L1, TGFβ-β, vaccine	CRC, melanoma, myeloid, pancreatic, prostate, solid	1, 1/2, 2
	FLT3 [68, 194–200]	CXD-301	0	11	CD40, PD-1, TLR3, chemotherapy, radiation, vaccine	Breast, H&N, lung, lymphoma, melanoma, prostate, solid	1, 1/2, 2
	STAT3 [201–208]	Danvatirsen, WP1066, TTI-101, OPB-111077, Napabucasin	4	9	Bcl-2, CTLA-4, DNMT, PD-L1, chemotherapy, radiation	Bladder, CNS, CRC, lung, lymphoma, melanoma, myeloid, pancreatic, solid	1, 1/2, 2, 3
	Siglec-15 [401–408]	NC318*	1	1	PD-1	Lung, solid	1/2, 2
	TREM2 [409–413]	PY314*	0	1	PD-1	Solid	1
Functional blockade	CD47 [209–215, 217, 218, 220, 221]	Magrolimab, Evorpacept, CC-90002, SRF231 Letaplimab, Lemzoparlimab, AO-176, TJ011133, SHR-1603, ZL-1201	3	18	Bcl-2, CCR4, CD20, CD38, DNMT, E3 ligase, GD2, PD-1, PD-L1, proteasome, VEGF-A, chemotherapy	GU, lymphoma, malignant heme, myeloma, myeloid, neuroblastoma, osteosarcoma, solid	1, 1/2, 2, 3
	SIRPɑ [209, 210, 212, 214, 216, 219, 222]	GS-0189, CC-95251, BI765063, TTI-621, TTI-622, Evorpacept, TG-1801, IMM0306, SL-172154, HX009, IBI322	10	21	Bcl-2, CD20, CD38, DNMT, E3 ligase, HER2, PD-1, proteasome, VEGFR2, chemotherapy, radiation	Cancer, CRC, esophageal, fallopian, gastric, H&N, lymphoma, malignant heme, myeloma, myeloid, ovarian, peritoneal, sarcoma, solid,	1, 1/2, 2, 2/3
Polarization	TLR9 [235–243]	CpG, CpG-STAT3 siRNA CAS3/SS3, CMP-001, Tilsotolimod, SD-101	1	16	4-1BB, ADT, BTK, CTLA-4, GnRH, OX-40, PD-1, PD-L1, radiation	Biliary, breast, CRC, hepatic, lymphoma, melanoma, pancreatic, prostate, solid	1, 1/2, 2
	CD40 [245–257]	Selicrelumab, Mitazalimab, CDX-1140, 2141-V11, Sotigalimab*, Dacetuzumab, Medi5068, ChiLob7/4, SEA-CD40, LOAd703, NG-350A	7	26	CD3, CSF1R, CTLA-4, EGFR, FLT3L, PD-1, PD-L1, TLR3, VEGF-A, chemotherapy, radiation, cellular therapies	Bladder, breast, CNS, CRC, esophageal, gastric, GI, lung, lymphoma, melanoma, ovarian, pancreatic, renal, sarcoma, solid,	1, 1/2, 2
	PI3Kγ [258–269]	Eganelisib*	1	3	PD-1, PD-L1, VEGF-A, chemotherapy	Breast, H&N, renal, solid	1, 2
	LILRB2 [425–430]	MK-4830, JTX 8064, IO-108	0	11	CTLA-4, PD-1, TIGIT, vaccine	CRC, lung, melanoma, renal, solid	1, 1/2, 2
	CLEVER-1 [431–435]	Bexmarilimab	2	1	PD-1	CRC, lung, renal, solid	1, 1/2
Metabolic reprogramming	CD73 [271–279]	Quemliclustat, LY3475070, Oleclumab, Mupadolimab, Sym021, IBI325, JAB-BX102, INCA00186, Uliledlimab*, NZV930, BMS-986179, HLX23, AK119, GS-1423	2	34	A2AR, A2BR, CTLA-4, EGFR, NKG2A, PD-1, PD-L1, VEGF-A, chemotherapy, radiation	Bladder, breast, CRC, lung, pancreatic, prostate, sarcoma, solid	1, 1/2, 2, 3
	CD39 [275, 277, 280, 281]	TTX-030, SRF617, IPH5201, ES002	0	5	A2AR, A2BR, CD73, PD-1, chemotherapy	Lymphoma, prostate, solid	1, 2
	A2AR [283–285]	Taminadenant, Ciforadenant, AZD4635*, Inupadenant	1	14	ADT, CD38, CD73, HIF2α, LAG3, PD-1, PD-L1, TIGIT, chemotherapy	Breast, lung, lymphoma, myeloma, prostate, renal, solid	1, 1/2, 2
	A2BR [283]	PBF-1129	1	1	PD-1	Lung	1
	A2AR A2BR [286]	Etrumadenant	0	11	ADT, CD39, CD73, IL-6, PD-1, PD-L1, TIGIT, VEGF-A, chemotherapy	CRC, H&N, lung, pancreatic, prostate	1, 1/2, 2
	IDO1 [287–291]	Epacadostat*, BMS-986205, KHK2455 navoximod, EOS200271, LY3381916, MK-7162	1	22	IL-15, LAG3, mTOR, PD-1, PD-L1, TGFβ-β, VEGF-A, chemotherapy, radiation, vaccine	Bladder, CNS, CRC, endometrial, fallopian, gastric, H&N, pancreatic, peritoneal, prostate, solid, urothelial	1, 1/2, 2, 3
Epigenetic reprogramming	HDAC [297, 299–309]	Entinostat*, Romidepsin, Tucidinostat, Mocetinostat, Domatinostat, Vorinostat*, Belinostat, Abexinostat, Panobinostat, Givinostat, Resminostat, Ricolinostat	17	147	AI, Bcl-2, BET, BTK, CD30, CD38, CDK 4/6, CTLA-4, DNMT, DRD2, E3 ligase, ER, GD2, GnRH, IL-2, IL-12, JAK, MEK, mTOR, NAE, ODC, PARP, PI3K, proteasome, PD-1, PD-L1, RT, TGFβ-β, TKI, VEGF-A, chemotherapy, radiation, cellular therapies	ALL, anal, bladder, breast, cervical, CNS, CRC, esophageal, fallopian, gastric, H&N, lung, lymphoma, melanoma, Merkel, MPN, myeloid, myeloma, neuroendocrine, ovarian, pancreatic, penile, peritoneal, prostate, renal, sarcoma, solid, urothelial, vulvar	1, 1/2, 2, 2/3, 3
Cytokines	STING [311, 312, 314–317]	BMS-986301, E7766, Ulevostinag, MK-2118, GSK3745417, TAK-676, SB11285, IMSA101 IACS-8803*, MIW815	1	9	CTLA-4, PD-1, PD-L1, radiation	Breast, H&N, lymphoma, solid	1, 1/2, 2
	IFNγ [319–322]	IFNγ 1b	1	2	HER2, PD-1, chemotherapy	Breast, lymphoma, myeloid, sarcoma	1, 1/2, 2
	IL-12 [323–333]	M9241*, GEN-1*, MEDI1191*, SAR441000	0	9	ADT, PARP, PD-1, PD-L1, TGF-β, chemotherapy, radiation	Breast, GU, lymphoma, ovarian, prostate, solid	1, 1/2, 2
	TNFR2 [334–343]	HFB200301, BI-1808, APX601, BITR2101, SIM0235	2	0	N/A	Solid	1, 1/2
	IL-1β [344–349]	Canakinumab, Anakinra, Gevokizumab	7	14	EPO, LAG3, PD-1, PD-L1, TIM-3, TKI, VEGF-A, VEGFR2, chemotherapy, radiation, cellular therapies	Breast, CRC, CLL, esophageal, gastric, lung, lymphoma, melanoma, myeloid, myeloma, pancreatic, prostate, renal	1, 1/2, 2, 3
	IL-6 [351, 353–356]	Tocilizumab, Sarilumab, Siltuximab*, Sirukumab, Olokizumab, Clazakizumab	0	10	CD3, CEA, CTLA-4, HER2, PD-1, PD-L1, chemotherapy, radiation	Breast, GU, lung, melanoma, pancreatic	1, 1/2, 2
	IL-8 [192, 193]	HuMax-IL8,	0	8	CDA, CTLA-4, DNMT, PD-1, radiation	H&N, hepatic, lung, myeloid, pancreatic, prostate, solid	1, 1/2, 2
	IL-10 [357–363]	Pegilodecakin	0	1	PD-1, TKI, chemotherapy	Solid	1
	TGFβ [364–370]	TASO-001, Galunisertib, Vactosertib, LY3200882, PF-06952229, AVID200, ABBV-151, SAR439459, NIS793, BCA101 trabedersen, ISTH0036, gemogenovatucel-T, belagenpumatucel-L, A83-01, SB-43-1542, RepSox, SM16, Bintrafup alfa, XPA-42-089	4	28	ADT, CD38, CDK 4/6, E3 ligase, IL-2, JAK, PD-1, PD-L1, TIM-3, VEGF-A, VEGFR2, chemotherapy, radiation	Breast, CNS, CRC, esophageal, hepatic, gastric, H&N, lung, MPN, myeloid, myeloma, ovarian, pancreatic, prostate, solid, urothelial	1, 1/2, 2, 3

*Indicates potentially significant results in clinical setting

## Targeting strategies against myeloid cells for cancer immunotherapy

In this section, we discuss ongoing strategies targeting the myeloid compartment in the preclinical and clinical settings which include: (1) altering myeloid cell composition within the TME through enhanced differentiation, proliferation, and recruitment of myeloid cells; (2) functional blockade of immune-suppressive myeloid cells; (3) reprogramming via either polarization, metabolic, or epigenetic modification of myeloid cells to acquire pro-inflammatory properties; (4) modulating myeloid cells via cytokines; (5) myeloid cell therapies; and (6) emerging targets such as Siglec-15, triggering receptor expressed on myeloid cells 2 (TREM2), macrophage receptor with collagenous structure (MARCO), leukocyte immunoglobulin-like receptor B2 (LILRB2), and common lymphatic endothelial and vascular endothelial receptor 1 (CLEVER-1) (Table 1).

### Strategies to alter myeloid cell differentiation, proliferation, and recruitment with the tumor microenvironment

In response to tumor-derived factors, immunosuppressive myeloid cells are consistently recruited, expanded, or differentiated to fuel tumor progression. One of the most straightforward strategies of targeting myeloid cells for cancer treatment is to alter the myeloid population composition, reducing the pro-tumor myeloid cell infiltration and increasing the abundance of anti-tumor immune cells. Strategies ranging from chemoattractant blockade to myeloid growth factors have been studied extensively in both preclinical animal models and clinical trials.

#### CCL2–CCR2 axis

The CCL2–CCR2 plays an integral role in the recruitment of myeloid cells including inflammatory monocytes, TAMs, and MDSCs. In metastatic CRC models, liver metastases which contain TAMs with high CCR2 expression are linked to a worse prognosis [165]. Inhibition of the CCL2–CCR2 axis suppresses tumor metastasis through reduced angiogenesis in preclinical models, in both direct manner, since CCL2 itself exerts an angiogenic effect, and indirect manner, which is through reduced chemoattraction of monocytes and macrophages [166–168]. A variety of inhibitors have been studied in the clinical setting to assess tumor response, which are summarized below.

Carlumab (CNTO 888) is a human monoclonal anti-CCL2 antibody with primarily negative clinical results. Carlumab was ineffective as monotherapy, as seen in a phase II study (NCT00992186) involving second-line therapy for metastatic castrate-resistant prostate cancer, where the objective response rate (ORR) was 0% and the median progression-free survival (mPFS) was only 2.7 months [135]. However, carlumab in combination with conventional chemotherapy (docetaxel, paclitaxel, carboplatin, gemcitabine, and PEGylated liposomal doxorubicin) for advanced solid tumors demonstrated improved clinical responses, including an ORR of 37.5% and a duration of response (DOR) of 6.3 months [169, 170]. Unfortunately, the effects of carlumab may be short-lived based on median CCL2 serum concentrations collected throughout the study period. While there was an initial reduction in total levels at the two-hour mark following initiation, there was a subsequent threefold to fivefold increase with further doses compared to baseline, regardless of the chemotherapy backbone, suggesting chemotherapy alone may have resulted in tumor response. Based on safety data, carlumab is well tolerated with the chemotherapy, with the most common grade 3 treatment-related adverse events (TRAEs) being cytopenias, fatigue, and stomatitis.

PF-04136309 is a small-molecule oral CCR2 inhibitor. In two small phase I trials (NCT01413022 NCT02732938), PF-04136309 was added to chemotherapy (FOLFIRINOX, nab-paclitaxel, and gemcitabine) in the management of advanced pancreatic cancer and produced response rates ranging from 23.8 to 48.5% [171, 172]. Pulmonary toxicity was reported in 24% when PF-04136309 was combined with nab-paclitaxel and gemcitabine. In the exploratory analysis, almost all recipients of PF-04136309 were found to have a decrease in peripheral blood CD14^+^ CCR2^+^ monocytes, though CCR2^+^ TAMs remained present in the majority of biopsy samples.

BMS-813160 is a small-molecule inhibitor that antagonizes both CCR2 and CCR5 and is currently under investigation in combination with nivolumab for the treatment of a variety of tumor types (NCT03496662, NCT03767582, NCT03184870, NCT04123379, and NCT02996110). Neither carlumab nor PF-04136309 has ongoing trials at this time.

#### CSF-1R

CSF-1 is a major lineage regulator and chemoattractant for TAMs. Preclinical data have demonstrated that inhibition of CSF-1R signaling repolarizes TAMs from M2-like to M1-like anti-tumor phenotype rather than simply depleting TAMs [173, 174]. One issue encountered with CSF-1R blockade has been compensatory upregulation of PD-L1 and CTLA-4 to maintain tolerogenic abilities, so clinical models have focused on a dual inhibitory approach involving CSF-1R blockade and ICIs to overcome this effect [175, 176].

While antagonists like sunitinib grossly block class III receptor tyrosine kinases (c-KIT, FLT3, CSF-1R, and PDGFR), dedicated CSF1R inhibitors have been developed, including small-molecule agents (pexidartinib, ARRY-382, BLZ945, and vimseltinib) and monoclonal antibodies (emactuzumab, cabiralizumab, IMC-CS4, AMG820, lacnotuzumab, PD-0360324, and axatilimab) [177, 178]. However, few have been able to demonstrate meaningful clinical activity. Two phase I trials involving LY3022855 monotherapy (NCT02265536, NCT01346358) and one phase I trial involving AMG 820 (NCT01444404) in the management of advanced solid tumors reported zero objective responses (0/86 and 0/25, respectively), though decreases in TAMs were noted in addition to elevations in circulation CSF-1 levels, indicating that proper target engagement occurred [179–181]. When LY302285 is used in combination with ICIs including tremelimumab (anti-CTLA-4) or durvalumab (anti-PD-L1), ORR approaches 4.2% (3/72) [182]. Similarly, for AMG820, when combined with pembrolizumab (anti-PD-1) for advanced solid tumors, ORR has been documented at 2.6% (3/116), well below expected response rates seen with pembrolizumab monotherapy [183].

One area of promise for CSF-1 inhibitors is in the management of tenosynovial giant cell tumors (TGCTs) and pigmented villonodular synovitis (PVNS) which are both rare, nonmalignant tumors that originate from the synovium of musculoskeletal joints and occur because of CSF-1 overexpression due to CSF-1/COL6A3 translocations [184]. Pexidartinib received FDA approval in 2019 following the results of the phase III trial (ENLIVEN) which randomized patients with unresectable TGCTs to receive pexidartinib vs. placebo. Following a 25-week follow-up period, the ORR was 38% (vs. 0% placebo, *p* < 0.0001) with a complete response (CR) rate of 15% [185]. Interestingly, ORR rates were similar between placebo crossovers and the initial pexidartinib arm, with crossover participants experiencing less hepatotoxicity, so the FDA did not include a loading dose in the approval [186]. Unique adverse events reported in ENLIVEN included changes to hair color (67%), transaminitis (39%), and nausea (38%), and both periorbital (13%) and peripheral (13%) edema among others.

#### CXCR1/2

The release of IL-8 by malignant cells and its subsequent binding to CXCR1 and CXCR2 on circulating myeloid cells and surrounding endothelial cells leads to the recruitment of MDSCs to the TME and the promotion of angiogenesis [187]. Ibuprofen inhibits IL-8 signaling, both through cyclooxygenase-2 (COX2) and non-COX2 pathways, and has been used as a base model for the development of novel CXCR1/2 inhibitors, including reparixin and ladarixin [188]. Other backbones have been explored as well, including nicotinamide antagonists (SX-682) and thiazolopyrimidine derivatives (AZD 5069).

Reparixin showed promising single-arm phase I trial data when combined with weekly paclitaxel in metastatic HER2-negative breast cancer (ORR 30%) [189]. However, subsequent randomized, two-arm data from the phase II fRIDA trial failed to detect a difference in the primary endpoint of mPFS when comparing the combination therapy to paclitaxel alone (5.5 vs. 5.6 months, respectively) [190]. Ladarixin is a second-generation dual inhibitor with stronger affinity for CXCR2, slowed melanoma progression in preclinical models, but clinical trials remain absent at this time [191]. Ongoing trials involving allosteric, reversible, small-molecule inhibitors SX-682 and navarixin as monotherapies and in combination with PD-1/PD-L1 agents are currently underway (NCT04245397, NCT03161431, NCT04599140, NCT04477343, NCT04574583, and NCT03473925).

Indirect methods of CXCR1/2 inhibition are also emerging, including the development of monoclonal antibodies which bind and sequester IL-8, such as HuMax-IL8 (BMS-986253) which has been shown in preclinical models to reduce PMN-MDSCs and prevent the mesenchymalization of TNBC [192]. Following a phase I study, HuMax-IL8 was found to provide no objective response as monotherapy, but multiple follow-up trials are ongoing involving its use in combination with immunotherapy agents (NCT04848116, NCT03689699, NCT02451982, NCT04050462, NCT03400332, NCT04572451, and NCT04123379) and chemotherapy (NCT05148234) [193].

#### FLT3L

FMS-like tyrosine kinase 3 receptor ligand (FLT3L) plays an active role in the maturation of macrophage-dendritic progenitors (MDPs) into pDCs and cDCs [194]. Preclinical studies have suggested that recombinant human FLT3 ligand (rhuFLT3L) agonism can lead to an enhancement in immunologic therapies, including PD-L1 inhibition [68]. Additionally, rhuFLT3L use has been shown to aid in the abscopal effect of radiation therapy by promoting immunogenic cell death [195, 196]. A similar abscopal effect has been noted when rhuFLT3L is combined with DC vaccine therapies [197]. Finally, in PD-L1 resistant mouse models, a combination approach involving FLT3L, radiotherapy, and TLR3/CD40 stimulation promotes CD8^+^ T cell influx, PD-L1 responsiveness, and tumor regression both locally and in distant untreated lesions, leading researchers to focus on this combination approach for clinical trials [198].

CDX-301 is a soluble rhuFLT3L developed using Chinese hamster ovary cells, and it has been shown to be a viable, well-tolerated option for combination trials [199]. Though it provides no clinical response on its own and public-domain clinical data remain scarce, preliminary phase II data (NCT0283925) involving CDX-301 in combination with single lesion SBRT resulted in 31% of analyzed subjects (9/29) recorded partial response (PR) involving distant lesions on PET imaging 2 months following therapy, further highlighting its abscopal potential [200].

#### STAT3

STAT3 has been implemented in immune escape and the promotion of tumor proliferation. The immunosuppressive potential of MDSCs occurs partially due to hindrances in myeloid progenitor differentiation as activated STAT3 inhibits the expression of protein kinase C βII (PKCβII) signaling [201]. Within the tumors themselves, constitutively activated STAT3 results in increased expression of PD-L1 along with the release of immunosuppressive cytokines (IL-6, IL-10, etc.) and growth factors such as CSF-1 and VEGF [202].

Considering STAT3 contributes to both tumor growth and the promotion of tolerogenic immune cells, it is an ideal target for cancer therapy development [203]. STAT3 activation occurs following phosphorylation by Janus kinases (JAKs) and subsequent homodimerization, leading it to translocate to the nucleus and perform its transcription functions. STAT3 and JAKs are then deactivated through Src homology domain-containing tyrosine phosphatases (SHP-1/2). While certain compounds have been found to impact STAT3 phosphorylation through drug repositioning studies (celecoxib, niclosamide, and pyrimethamine) or through known JAK inhibitors (ruxolitinib and pacritinib), more selective STAT3 inhibitors have since been developed including small-molecule inhibitors (napabucasin, TTI-101, OPB-51602, OPB-31121, OPB-111077, BP-1-102, and S3I-201) and oligonucleotides (danvatirsen and STAT3 DECOY) [202, 204, 205].

While the majority of trials (NCT02753127, NCT02993731, NCT01839604, NCT00955812, NCT00657176, NCT01406574, NCT01344876, NCT01711034, NCT02178956, NCT02315534, and NCT02279719) have failed to document meaningful clinical efficacy, as monotherapy or in combination (FOLFIRI, gemcitabine, paclitaxel, sorafenib, and temozolomide), several agents that have off-target effects that lead to lower STAT3 activity are currently being explored, including SHP-1/2 agonists like SC-43 [NCT04733521] and IL-6R inhibitors like tocilizumab (NCT02767557, NCT04940299, and NCT04691817) and siltuximab (NCT04191421) [202, 206–208].

### Strategies to functionally block immune-suppressive myeloid cells

#### CD47-SIRP⍺

CD47 is ubiquitously expressed on the surface of normal tissue in order to allow for immune self-recognition. This occurs when CD47 binds to SIRP⍺ which is found on macrophages and DCs [209]. Tumor cells take advantage of this system via overexpression of CD47, providing a unique immune escape mechanism that has garnered considerable interest. Within TAMs, SIRPα expression also remains high and binding to CD47 within the TME further assists TAMs in maintaining their immunosuppressive phenotype through SHP-1/2 signaling [210]. Preclinical studies have found that antagonizing CD47/SIRPα signaling results not only in augmented phagocytosis, but also in DC activation, CD8^+^ T cell priming, and a decrease in myeloid-driven immunosuppression through macrophage polarization and an increased M1 to M2 ratio [211, 212]. Unique inhibitors of the CD47-SIRP⍺ axis include monoclonal antibodies against CD47 (magrolimab also known as Hu5F9-G4, evorpacept, CC-90002, SRF231, letaplimab, lemzoparlimab, AO-176, TJ011133, SHR-1603, and ZL-1201), monoclonal antibodies against SIRP⍺ (BI765063, GS-0189, CC-95251), and recombinant SIRP⍺-Fc fusion proteins (TTI-621, TTI-622, and evorpacept) [213, 214]. Bispecific antibodies are also emerging with secondary targets including CD19 (TG-1801), CD20 (IMM0306), CD40L (SL-172154), PD-1 (HX009), and PD-L1 (IBI322) [215].

Developing a monoclonal antibody toward SIRPα can be challenging considering that various SIRP homologs exist alongside various SIRPα alleles, so agents require pan-allele sensitivity while avoiding SIRP homolog activity [216]. Advantages, however, include the fact that SIRP is not ubiquitously expressed, allowing for anti-SIRPα therapies to avoid the destruction of bystanders such as red blood cells, as seen with anti-CD47 agents. This also allows them to be given at lower doses while theoretically maintaining efficacy due to decreased antigen sink. Many of the monoclonal anti-CD47 agents currently developed target different epitopes and as a result, a specific subset has been found to only weakly bind to red blood cell CD47 (lemzoparlimab, magrolimab, and AO-176), allowing them to spare these cells and prevent the development of anemia [217]. Additionally, newer anti-CD47 agents have been developed with inert Fc regions (evorpacept) to further avoid this effector function, though as a result these therapies become reliant on combination therapies involving a tumor-opsonizing antibody [218]. The SIRPα-Fc fusion products are made up of IgG Fc fused to the extracellular domain of SIRPα and this structure allows for SIRPα to bind to CD47 for a longer duration by slowing clearance through the presence of the Fc domain [219]. Though affinity for native SIRPα may be lower compared to anti-CD47 mAbs, SIRPα variants have been designed to overcome this deficiency. The small molecular weights seen with these fusion proteins may also assist with their ability to penetrate TME more readily. Bispecific antibodies aim to provide dual-signaling and improve immune cell proximity, though whether this correlates to improved efficacy remains to be seen.

The most promising clinical data involve the use of magrolimab in combination with rituximab ± chemotherapy (gemcitabine and oxaliplatin) for the treatment of relapsed/refractory B cell non-Hodgkin lymphoma (NHL), where researchers noted an ORR of 50% (11/22) with CR noted in 36% (8/36) of participants (NCT02953509) [220]. Contrast this to the phase I results involving magrolimab monotherapy in the treatment of advanced solid tumors where the ORR approached 5% (NCT02216409, NCT30811285) [221]. Similarly, evorpacept in combination with pembrolizumab ± trastuzumab for advanced solid tumors (ASPEN-01 and NCT03013218) resulted in an ORR of 0% (0/15) and a disease control rate (DCR) of 26.7% (4/15) [222]. Biopsies obtained from participants post-treatment showed increases in TAM populations on immunohistochemistry staining, and no increase in CD8^+^ tumor-infiltrating lymphocytes (TILs) was noted in either treatment arm.

#### CD24-Siglec-10

CD24 suppresses inflammatory responses through binding to sialic acid-binding immunoglobulin-type lectin-10 (Siglec-10) found on the surface of macrophages [223]. However, CD24 has also recently been found to provide a unique immune escape mechanism utilized by a variety of cancer cells [224]. Though CD24 is primarily expressed on immune progenitor cells and lymphoid tissue, certain tumor types have been found to express CD24 at high magnitudes [224, 225]. To elicit an effect, CD24 binds to TAMs via surface-bound Siglec-10, resulting in immune escape through SHP-1 and SHP-2 signaling, similar to CD47. To put this theory of immune escape to the test, researchers removed the CD24 protein gene from human breast cancer cell lines, then intermixed these CD24-deficient cells with wild-type cancer cells. They confirmed that macrophages cleared out the CD24-deficient populations more rapidly [224]. These cells were also significantly more sensitive to anti-CD47 therapies, suggesting a plausible synergistic role with some of the CD47-targeting agents mentioned previously. Finally, Siglec-10 knockout macrophages were also created, resulting in improved phagocytosis abilities compared to controls.

CD24 also plays a potential role in cancer migration in various cancer types along with prognostication [226, 227]. As a result, many preclinical studies now closely evaluate targeting this signaling pathway as a way of combating both malignancies and the TME. Initial models involved unconjugated monoclonal antibodies targeting the leucine–alanine–proline (LAP) epitope of CD24 (SWA11) which led to antibody-dependent cellular cytotoxicity (ADCC) in lung, ovary, bladder, myeloma, and lymphoma models, all while notably altering the cytokine milieu and hindering metastatic potential [228]. Bispecific antibodies involving MHC-I (cG7-MICA) and CD30 have also been examined with similar results reported. Success has also been noted with antibody–drug conjugates involving various payloads including nitric oxide, pseudomonas exotoxin, and even ricin A-chain immunotoxin [228–231]. More recently, anti-CD24 chimeric antigen receptor (CAR) T cells and NK products have been investigated in pancreatic and ovarian cancer models with the use of CARs derived from SWA11, with dual targeting seeming to help reduce the incidence of off-target events [228, 232, 233].

A humanized, affinity-matured version of anti-CD24 has already been developed (ONC-781) and this monoclonal antibody has been used to construct an antibody–drug conjugate (ONC-784), a bispecific antibody to CD3 (ONC-783), and a CAR-T therapy (ONC-782) for potential clinical trials [234]. Little remains publicly available regarding clinical trial prospects, but it seems fair to say that dual inhibition of immune escape mechanisms (PD-L1, CD47, and CD28) will likely be on the horizon.

### Strategies to reprogram myeloid cells to acquire pro-inflammatory properties

#### TLR agonists

Sensing of DAMPs and PAMPs through TLRs expressed by APCs results in their activation and subsequent T cell priming [235]. TLR agonists are studied as adjunct therapies to tumor vaccines and immunotherapy agents to amplify treatment response. However, modifications of TLR agonists are required for clinical use to adjust for their short half-life, poor localization, and limited immunogenicity [236]. For the purpose of this review, we will be discussing TLR9 agonists which have been the most extensively studied TLR agonists within the clinical trial setting.

TLR9 is constitutively expressed within the endosomes of B Cells and pDCs, though additional myeloid subtypes have been found to express TLR9 when activated by immune triggers including infection [235]. TLR9 recognizes unmethylated cytosine-phosphate guanine (CpG) oligodeoxynucleotides (ODNs) found on modified or foreign DNA, resulting in robust activation of innate and adaptive immune cells through MyD88 signaling [237]. This discovery has led researchers to engineer TLR9 agonists based on CpG ODNs. Given these agonists are physiologic derivatives, they naturally carry shorter half-lives, but with modifications including a nuclease-resistant phosphonothioate backbone (CPG 7909, ISS 1018, CpG-28, IMO-2055, tilsotolimod, SD-101, GNKG168, and S-540956), the half-life of these agents has been increased from minutes to days [237]. Other modifications include the creation of double stem-loop immunomodulators (dSLIMs) which are CpG DNA molecules that have been covalently closed, forming a dumbbell-like shape that is resistant to DNase degradation (lefitolimod and EnanDIM) [238]. Additionally, various delivery vehicles have been explored to improve localization and bioavailability including nanoparticles (cavrotolimod) and viral-like particles (CMP-001 and NZ-TLR9) [238]. TLR9 agonists are also being investigated as conjugate payloads as part of antibody–drug conjugates for monoclonal antibodies, including anti-SIRPα (ALTA-002) and anti-CD22 (TAC-001). These have been collectively termed as “Toll-like receptor agonist antibody conjugates” (TRAAC) [239].

Preclinical data involving modified CpG ODNs in murine models have demonstrated that intratumoral injections result in tumor regression along with tumor-specific T cell responses and upregulation of immune checkpoint genes including PD-L1, OX40, and CTLA4 [240]. This has been a key justification for combining checkpoint inhibitors with CpG ODNs. Additionally, CpG ODNs are radiosensitizers in early lung cancer models with a sensitivity enhancement ratio (SER) of 1.28, further justifying a multi-therapy approach [241]. In the clinical setting, single-arm phase II results involving intratumoral injections of a CpG agonist (PF-3512676) plus local radiation in low-grade B cell lymphoma noted an ORR of 23.3% (7/30) with a DCR of 86.6% (26/30) [242]. Ongoing phase I studies are examining the use of CpG ODNs in combination with local radiation and immunotherapy agents for the management of refractory lymphomas (NCT03410901).

PF-3512676 (CPG 7909) is the most extensively studied clinical CpG ODN, particularly in combination with conventional chemotherapy (paclitaxel, carboplatin) for the treatment of NSCLC. Initial phase II trials appeared promising with improvements in OS compared to chemotherapy alone but following the release of interim results from two phase III trials, both trials were terminated due to high rates of sepsis-related events and minimal evidence of improved clinical efficacy [243]. CpG ODNs continue to be studied as adjuncts, particularly in the realm of cancer vaccine therapies given their immunostimulatory properties.

#### CD40 agonists

CD40 is readily expressed on antigen-presenting cells and is essential to their activation. Additionally, its ligand CD40L is found on a variety of immune and non-immune cells, including CD4^+^ T cells. CD40L helps with the cross-priming of CD4^+^ cells to non-self-antigens by providing a co-stimulatory effect [244]. Activation of CD40 on DCs leads to upregulation of MHC molecules, increase in IL-12 secretion, and the promotion of cytotoxic T cell activation [245]. Preclinical mouse models and pilot human studies involving CD40 agonist antibodies in combination with gemcitabine in the treatment of pancreatic cancer have shown that CD40 activation helps reverse immunosuppression with modest tumor response rates [246].

Recently developed CD40 agonists include fully human IgG monoclonal antibodies (selicrelumab, mitazalimab, CDX-1140, and 2141-V11), humanized IgG monoclonal antibodies (sotigalimab also known as APX005M, SEA-CD40, and dacetuzumab), chimeric IgG antibodies (ChiLob7/4), recombinant CD40L fusion proteins (MEDI5083), and vaccine-delivered transgenes (LOAd703 and NG-350A) [247]. One feature that separates the monoclonal antibodies apart is their antibody isotype, with most IgG1 models needing FcγR cross-linking to produce a signal (sotigalimab, ChiLon7/4, ADC-1013, and SEA-CD40) whereas IgG2 antibodies mimic CD40L signaling independent of FcγR cross-linking [245, 248]. Additionally, newer IgG1-based monoclonal antibodies have modified (non-fucosylated) Fc regions which help increase their affinity to FcγR in an attempt to improve ADCC (SEA-CD40 and APX005M). Another separating feature for antibodies is epitope binding, with studies showing that agonistic activity decreases for a given antibody as its epitope target draws closer to the cellular membrane, often leading to the development of antagonistic properties [249].

Overall, tumor response rates with single-agent CD40 monoclonal antibodies have been low to date. Single dose selicrelumab was able to produce an ORR of 27% (4/15) in melanoma participants, but in a separate trial involving weekly selicreulmab for advanced melanoma, the ORR was 0% (0/11) with evidence of T cell depletion observed in the exploratory analysis [250, 251]. In combination with tremelimumab for treatment-naive metastatic melanoma, selicreulmab provided an ORR of 27.3% (6/22) with a CR rate of 9.1% (2/22) and evidence of increased T cell infiltration and activation [252, 253]. This is an improvement when compared to separate tremelimumab monotherapy phase III trials where treatment-naïve patients with metastatic or unresectable melanoma achieved an ORR of only 10.7% (36/328) and a CR of 3% (11/328) [253].

Similarly, a phase I study of sotigalimab (APX005M) combined with nab-paclitaxel, gemcitabine, and PD-1 blockade (nivolumab) in metastatic pancreatic adenocarcinoma produced a promising ORR of 58% (14/24), though there were two treatment-related deaths attributed to sepsis (8.3%) [254]. Without sotigalimab, a separate phase I trial involving nab-paclitaxel, gemcitabine, and nivolumab in treatment-naïve stage IV pancreatic adenocarcinoma noted an ORR of only 18% [255].

Contrast these results to a phase 1b solid tumor trial involving selicreulmab in combination with atezolizumab which found an ORR of only 10% (8/80), though CD8^+^ T cell activation expansion was documented, and all responses were linked to subcutaneous dosing over IV dosing [256]. Finally, the use of dual TAM polarizing agents (sotigalimab and cabiralizumab) with or without nivolumab in NSCLC in the phase I setting resulted in no responses but did increase pro-inflammatory cytokine levels along with CD40 expression [257]. Though initial clinical data are underwhelming, further optimization of dosing frequencies and sequencing may help improve efficacy in subsequent studies.

#### PI3Kγ inhibitors

Phosphatidylinositol 3-kinase gamma (PI3Kγ) activation aids in the polarization of TAMs into the M2-like phenotype. The use of a PI3Kγ inhibitor reverses this partially due to the upregulation of IFNγ which signals TAMs to revert back to an M1 phenotype, thereby promoting anti-tumor immunity [258]. This has been documented in PI3Kγ^−/−^ pancreatic murine models where blockade of PI3Kγ leads to TAM reprogramming and improved cytotoxic T cell mobilization into the TME [259]. PI3Kγ inhibitors also provide synergy when combined with anti-PD-L1 therapy in the realm of HNSCC which tends to be immunologically inert [260]. PI3K inhibitors vary based on their affinity to the four main class I PI3K isoforms: alpha, beta, delta, and gamma. While preclinical hematologic models have suggested that pan-PI3K inhibitors may provide modest improvements to cytotoxic potential compared to dual inhibitors, PI3K inhibition is often plagued with toxicities that limit their clinical utility, making selective inhibitors a desirable option in hopes of improving treatment tolerability [261]. While hyperglycemia has been linked more so to PI3Kα inhibition, whereas rates of severe colitis and pneumonitis are higher with PI3K-δ and PI3Kγ dual inhibitors (idelalisib and duvelisib) [262, 263].

Selective PI3Kγ inhibition is relatively new following the emergence of eganelisib, though others are currently in development with promising PI3Kγ affinity (AZD3458) [264]. Preclinical murine studies primarily focused on TNBC, melanoma, CRC, and lung models have shown that eganelisib reverts TAMs back to a M1 phenotype with increased IL-12 and iNOS levels. Additionally, combining eganelisib with both anti-CTLA4 and anti-PD-1 therapy results in CR rates of 30% in breast and 80% in melanoma models (B16-GM-CSF) and provided immunity to tumor re-implantation, whereas dual checkpoint inhibition alone did not result in any complete responses. This ultimately led to a phase I trial (MARIO-1) which involved eganelisib as monotherapy and in combination with nivolumab (anti-PD-1) for the treatment of advanced solid tumors [265]. Data from the melanoma and HNSCC expansion cohorts were later presented with combination therapy providing an ORR of 7.7% (3/39) and 10.0% (2/20), respectively, with a favorable safety profile and translational data demonstrating decreases in measured MDSC levels [266, 267]. In MARIO-3, eganelisib was combined with atezolizumab and nab-paclitaxel as first line therapy for TNBC with interim results including an ORR of 56.1% (23/41) in the intention-to-treat, an ORR of 48.1% (13/27) in PD-L1-negative participants, and a DCR of 81.4% (22/27) [268]. Researchers then compared survival outcomes to that of IMpassion130 as a historical control, with PD-L1 patients having an mPFS of 11.0 months (vs. 7.5 months), while PD-L1-negative patients carried an mPFS of 7.3 months (vs. 5.6 months). Only 14% of patients (n = 7) discontinued treatment due to adverse events, including hepatotoxicity, peripheral neuropathy, and rash. Finally, translational biopsy data documented increased PD-L1 expression at 2 months post-treatment, resulting in 5 out of 8 sampled PD-L1-negative tumors and surrounding immune cells converting to a PD-L1 positive status. Additional eganelisib trials remain underway, including MARIO-275, a phase II trial comparing nivolumab monotherapy to combination therapy in urothelial cancer, with initial data reporting an ORR of 30.3% (10/33) in the experimental arm versus 25% (4/16) with nivolumab alone and no notable difference in mPFS between arms at this time (9.1 vs. 8.0 months, HR 0.79, 95% CI 0.39–1.60) [269].

#### CD39/CD73/A2AR/A2BR

Apoptotic and hypoxic cells often release high amounts of adenosine triphosphate (ATP) into the extracellular domain, which in turn can signal a cascade of inflammatory responses. This occurs as a result of ATP binding to purinergic receptors P2X and P2Y, triggering inflammasome activation and neutrophil chemotaxis, respectively [270]. In order to counteract this inflammatory response, ATP is enzymatically broken down by enzymes CD39 and CD73 which are highly expressed on the surface of MDSCs and tumor cells [271]. CD39 converts ATP to adenosine monophosphate (AMP) whereas CD73 converts AMP into adenosine. Adenosine then acts as a powerful immunosuppressive metabolite through binding to adenosine receptors found on immune cells such as A2AR and A2BR, further inducing immunodormant states among TAMs, neutrophils, DCs, and MDSCs alike while also promoting Treg differentiation [272–274]. Given the malignant and tolerogenic nature of CD73, CD39, A2AR, and A2BR, numerous inhibitors have been developed for each target, including dual inhibitors of the targets above [275–277].

Regarding CD73, small-molecule inhibitors (quemliclustat and LY3475070), monoclonal antibodies (oleclumab, mupadolimab, Sym024, IBI325, JAB-BX102, INCA00186, NZV930, BMS-986179, HLX23, AK119, and uliledlimab), and bispecific antibodies (TGFβ: GS-1423) currently crowd the pipeline [275, 276]. Among the monoclonal antibodies, oleclumab has been the most widely investigated. This humanized IgG1 non-Fc-binding anti-CD73 antibody appears to act as an allosteric inhibitor and has been shown to have a picomolar affinity to CD73, but it comes with a few drawbacks. Initial phase I trial data involving oleclumab monotherapy remain unpublished, but in combination with durvalumab (anti-PD-L1), several trials were withdrawn due to reportedly low ORRs and one phase II trial involving ovarian cancer participants reported a DCR of only 27% [278]. Regarding newer iterations, interim data released from a phase I trial involving uliledlimab in combination with atezolizumab (anti-PD-L1) in the management of advanced solid tumors reported an ORR of 23% (3/13) with a DCR of 46% (6/13), and a significant trend toward increased CD73 expression among treatment responders compared to non-responders (78% vs. 23%) [279].

For CD39 inhibitors, the selection is less robust, with the majority of available agents being monoclonal antibodies that remain in early-stage clinical trials (TTX-030, SRF617, IPH5201), though others are currently under development (ES002) [275, 277]. The anti-tumor activity with IPH5201 has been shown in animal models involving human CD39 knock-in mice injected with melanoma cell lines (B16F10) with researchers able to link the blocking of ATP hydrolysis through inhibition of both membrane and soluble CD39 to the subsequent activation of TAMs and DCs [280]. Additionally, in human CD39 knock-in models, IPH5201 attenuated the anti-tumor activity of chemotherapy agents like oxaliplatin which cause effluxes of ATP from tumor cells. Antisense oligonucleotides are also under development using a locked nucleic acid methodology that allows for blockage of CD39 mRNA. To date, preclinical data suggest that following a dose-dependent suppression of CD39 mRNA expression in tumor-bearing mice, CD8^+^ T cell expansion shortly follows along with increases in PD-1 positive TIL expression and drops in Tregs, TAMs, and CD39 protein levels [281]. Dual inhibition with anti-PD-1 antibodies has been shown to further inhibit tumor within these murine models.

Finally, attention has been placed on inhibiting adenosine signaling through antagonism toward receptors found on immune cells, including A2AR on T cells, MDSCs, TAMs, DCs, and A2BR on NK cells, MDSCs, TAMs, and DCs. These agents have already been shown to be quite tolerable among vulnerable patient populations, including those with Parkinson's disease, as they have been linked to regulating dopamine signaling [282]. Among these agents include selective A2AR inhibitors (taminadenant, ciforadenant, AZD4635, inupadenant, and preladenant), selective A2BR inhibitors (PBF-1129), and dual inhibitors (etrumadenant) [283]. The A2AR antagonist AZD4635 has been studied as monotherapy in CRC murine models CT26 and MC38 and has been shown to slow tumor growth by 44% and 73%, respectively, with improvements to 73% and 91% with the addition of anti-PD-1 therapy [284]. AZD4365 also increases the presence of intratumoral CD103^+^ DCs and OVA antigen-specific CD8^+^ T cells. Clinical trial data are limited, but a similar inhibitor, ciforadenant, has been shown in phase I studies to provide an ORR of 8% (2/25) and a DCR of 60.0% (15/25) cumulatively in patients with renal cell carcinoma (RCC) and prostate cancer [285]. As for A2BR inhibitors, there is an ongoing phase I trial evaluating PBF-1129 in lung cancer (NCT03274479) but results are yet to be released. Finally, for dual inhibitors, early results from phase I studies involving etrumadenant (AB928) combined with anti-PD-1 therapy have shown linear pharmacokinetics, moderate tolerability (1 DLT, grade 2 rash), and an ORR of 8.3% (1/12) with a DCR of 33.3% (4/12) [286].

#### IDO1 inhibitors

As discussed above, myeloid cells including TAMs, DCs, and MSDCs express high levels of the enzyme IDO1, which is important for the degradation of L-tryptophan into kynurenine [287]. The subsequent depletion of L-tryptophan from the TME has been linked to the arrest of cytotoxic T cells [288]. Tumor draining lymph nodes tend to be the areas of highest IDO1 expression, particularly on the surface of APCs, with research suggesting this expression contributes to a tumor’s ability to form locoregional metastases, as seen in breast models [287, 289].

IDO inhibitors are primarily composed of small-molecule inhibitors such as epacadostat, navoximod, BMS-986205, EOS200271, KHK2455, LY3381916, and MK-7162. The first IDO inhibitor to advance through early-phase trials was epacadostat, eventually ending up in a phase III randomized, international, placebo-controlled trial as a therapy for unresectable stage III & IV melanoma in combination with pembrolizumab to assess whether it improved immune checkpoint efficacy [290]. Researchers reported no meaningful difference in terms of mPFS (4.7 vs. 4.9 months with placebo, HR 1.00), ORR (34 vs. 32%), CR (4% vs. 4%), DCR (51% vs. 51%), or treatment-related adverse events (10% vs. 9%), with 72–73% of participants having a positive PD-L1 status and 62–66% having a positive IDO1 status. While some developers have pivoted toward IDO1 inhibitor modifications to improve efficacy, others have set their sights on additional tryptophan metabolism pathways. Similar to IDO1, tryptophan-2,3-dioxygenase (TDO) serves the same function of degrading the L-tryptophan, into N-kynurenine, but what separates TDO from IDO1 is its expression patterns, with a higher predominance seen within the liver, bone marrow, brain, immune system, genitourinary tract, and gastrointestinal tract [291]. Like IDO1, TDO has been linked to immune resistance, including in mouse models where, in the presence of TDO inhibition, immune sensitivity was restored in those injected with TDO-expressing cancers. Within this same study, researchers also demonstrated that across multiple human cancer types, 32% expressed IDO1 alone, 35% expressed TDO alone, and 51% expressed both markers, making a case for dual IDO1/TDO inhibition. Currently developed IDO1/TDO inhibitors include HTI-1090, DN1406131, RG70099, and EPL-1410. Another avenue of active research includes the targeting of downstream signaling proteins, including aryl hydrocarbon receptor (AHR) which has been linked to Treg activation through kynurenine, along with other kynurenine metabolism enzymes such as KATI/II/III, KYNU, and KMO [292].

#### HDAC inhibitors

While certain DNA sequences may remain preserved within cancer cells, their expressional patterns can vary considerably depending on the presence of epigenetic modifications, including noncoding RNAs, DNA methylation, and histone modifications [293]. These changes have been linked to the metastatic potential of cancer cells as they continue to evolve, making these posttranslational modifications a key hallmark of malignancy [294]. Cellular metabolism plays a key role in the activity of certain HDAC that have been linked to immunosuppressive functions [295]. While certain HDACs (class III) require nicotinamide adenine dinucleotide (NAD^+^) as a cofactor, which is a byproduct of anaerobic glycolysis, histone acetyltransferases (HATs) require acetyl-CoA, the end product of aerobic glycolysis, in order to reverse these effects. This skewed ratio of NAD^+^ to acetyl-CoA within the TME further aids in the transformation of tumor cells and immune cells alike. HDAC activity within TAMs has been linked to decreased MHC-II expression, as seen in murine cancer models [296]. Additionally, HDAC has been shown to lessen MHC-I expression within cancerous cells to prevent them from presenting tumor-associated antigens to immune cells [297]. Both HDAC effects have been shown to be reversible with the introduction of an HDAC inhibitor (HDACi), resulting in tumor cell destruction [296–298]. Finally, HDAC inhibitors have been shown to deplete MDSCs within in vitro tumor models, further justifying their use as an immunotherapy adjunct [299].

Classical human HDAC enzymes tend to be zinc-dependent and come in various classes, with class I being ubiquitously expressed (HDAC1, HDAC2, HDAC3, and HDAC8), whereas class II (HDAC-4, HDAC-5, HDAC-6, HDAC-7, HDAC-9, and HDAC-10) and class IV (HDAC-11) have expression limited to cells of the central nervous system and muscular cells [300]. When it comes to HDAC inhibitors, most tend to have activity against class 1 HDACs (entinostat, romidepsin, mocetinostat, domatinostat, valproic acid, and phenylbutyric acid), though pan-inhibitors exist (panobinostat, abexinostat, givinostat, resminostat, quisinostat, pracinostat, belinostat, and vorinostat) as do more selective inhibitors (ricolinostat—HDAC6) [301, 302]. Vorinostat was the first dedicated HDACi to get propelled into clinical trials, particularly in the management of acute myeloid leukemia (AML). However, a phase II study of vorinostat as monotherapy in the management of high-risk AML published a CR of 2.7% (1/37), vastly underperforming the standard 40% CR rate seen with conventional therapies at the time [303]. Similarly, in a phase II randomized trial comparing azacitidine monotherapy to azacitidine plus vorinostat in AML found no difference in ORR (41 vs. 42%), CR (22% vs. 26%), or OS (9.6 vs. 11.0 months, *p* = 0.32) between the control and experimental arms, respectively [304]. Despite initial discouraging results, vorinostat rebounded as a potential lymphoma therapy based on small-scale phase I data. Eventually, two simultaneous phase II trials investigating vorinostat monotherapy in those with refractory cutaneous T cell lymphoma (CTCL) led to its FDA approval after investigators reported a cumulative ORR of 28.0% (30/107) with a median time to progression of 148 and 212 days for each study [305]. This was followed by FDA approval of romidepsin and belinostat for similar findings of durable treatment response in multicenter phase II trials.

For the majority of solid tumor types, however, results have been underwhelming. Single-agent HDACi therapy has failed to induce a partial or complete response in the vast majority of phase I and II trials involving HNSCC, breast cancer, thyroid cancer, ovarian cancer, and glioblastoma multiforme [306]. One phase II trial explored vorinostat in relapsed or refractory solid tumors including breast, CRC, and NSCLC with no reported responses and 68.8% (11/16) of participants discontinuing therapy due to adverse events including diarrhea, nausea, thrombocytopenia, fatigue, and anorexia [307]. One area of hope for HDACi therapy involves their use as adjuvant agents, particularly in combination with immunotherapy, as in vivo models of immune-resistant breast and pancreatic cancer have shown that the use of an HDACi-like entinostat can weaken MDSC-suppressive functions and improve CD8^+^ effector T cell activity compared to checkpoint therapy alone [308].

### Strategies to modulate myeloid cells via cytokines

#### Type I interferons: IFNα and IFNβ

One of the first cytokines to be directly linked to anticancer activity is IFNα, a type I IFN produced many cells but most abundantly by the pDCs. IFNα is constitutively expressed in most cells and its production becomes pronounced when cells detect aberrant intracellular DNA or RNA, such as that seen in tumors or viral infection [81]. Activation of the cytosolic nucleic acid sensing pathways or TLRs leads to type I IFN and pro-inflammatory production, such as IL-12, TNFα, CXCL9, and CXCL10, that are important for T cell trafficking and function [309].

Using recombinant DNA technology, researchers were able to create the first FDA-approved immunotherapy against cancer, recombinant IFNα2 (rIFNα2), which received its approval in 1986 based on a single-arm, multicenter trial involving refractory hairy cell leukemia patients [310]. In the decades following, recombinant IFNα2 faded off into obscurity due to the development of more efficacious alternatives. Additionally, the tolerance of IFNα and IFNβ analogs has historically been poor, both due to flu-like symptoms and inconvenient dosing schedules. This led to the development of STING agonists, which mimic the physiologic cyclic dinucleotide (CDN) molecules cyclic 2’,3’-cGAMP, a product of cGAS [311]. Momentum for STING agonists grew after preclinical studies involving STING-deficient tumor-bearing mice were demonstrated as having fewer IFNγ-producing CD8^+^ T cells with increased Tregs and MDSCs [312]. Furthermore, cGAS deficient mice fail to mount a response to PD-L1 therapy compared to wild-type counterparts in a murine melanoma model [313]. Taken a step further, STING agonists like IACS-8803 have been shown to reverse these effects by repolarizing suppressive myeloid subsets in both human and mouse pancreatic cancer models, leading to increased sensitivity of orthotopic cancer cells to checkpoint inhibitor therapy [314].

One of the earlier STING agonists to reach large-scale clinical trials was vadimezan. A phase III trial investigating carboplatin and paclitaxel with or without vadimezan in advanced NSCLC observed the same median PFS (5.5 vs. 5.5 months) and ORR (25 vs. 25%) in both arms, but with a higher rate of grade 4 neutropenia (27% vs. 19% with control, respectively) and infusion site reactions (10% vs. 0.5%) [315]. Newer versions of STING agonists have been modified to improve STING affinity, cell permeability, and resist hydrolysis through ENPP1 (ectonucleotide pyrophosphatase/phosphodiesterase family member 1) [311]. Current STING agonists under clinical investigation include small-molecule agonists like MIW815, BMS-986301, E7766, ulevostinag, MK-2118, GSK3745417, TAK-676, SB-11285, and IMSA-101 [316]. In addition, small-molecule inhibitors of ENPP1 (MAVU-104, MV-626) have been developed as an off-target method of activating STING, with clinical trials already up and running [311, 316]. Finally, newer delivery methods for STING agonists are being explored, including STING agonist antibody–drug conjugates (CRD5500), exosomes (exoSTING), liposomal nanoparticles (STING-NPs), and vaccines (ONM-500 nanovaccine) [317].

#### Type II interferons: IFNγ

IFNγ is a type II interferon which is paramount to enhancing adaptive and innate cytotoxic activity. IFNγ exerts this effect of macrophages through JAK/STAT signaling, leading to activation of ISGs which control the production of inflammatory cytokines while also increasing MHC molecule and phagocytic receptor expression on macrophages, thereby activating them [318]. Within the TME, these actions by IFNγ promote the conversion of M2-like TAMs into M1 phenotypes, causing them to incite anti-tumor activity. However, IFNγ production and secretion from T cells and NK cells often requires interactions with M1 macrophages, hence why IFNγ remains low within the TME given the lack of a catalyst. Additionally, with extended periods of inflammation, IFNγ can activate negative feedback signals, leading to the increased PD-L1 expression and IDO1 expression on TAMs, which in turn inhibit NK cell activity [319, 320].

Using IFNγ as a therapeutic agent comes with unique challenges, including concerns for systemic cytotoxicity. This was best illustrated in a phase III trial involving advanced stage ovarian cancer patients where participants were randomized to receive chemotherapy with or without recurring subcutaneous IFNγ 1b injections [321]. The trial was stopped early due to a significantly higher mortality rate in the IFNγ 1b arm compared to placebo (39.7% vs. 30.4%, respectively) which appeared to be driven by higher rates of serious cytopenias (34.5% vs. 22.7%, respectively) and fever (20.6% vs. 5.0%). Despite these findings, given the link between IFNγ expression and its link to PD-L1 expression, a resurgence of interest has taken place, with multiple clinical trials involving recombinant IFNγ alongside anti-PD-1/PD-L1 agents (NCT02614456 and NCT03063632). Drug development has also geared toward incorporating IFNγ as an adjunct to vaccine therapies [322].

#### IL-12

APCs are the main source of IL-12, a vital pro-inflammatory cytokine that boosts IFNγ and TNFα production from cytotoxic cells and promotes effector cell activation. In the setting of malignancy, IL-12 can also play an important role in reprogramming MDSCs and TAMs [323, 324]. Through effector cell promotion, IL-12 therapy has been shown to guide anti-tumor activity in a wide variety of preclinical models, including lymphoma, renal cell, breast, ovarian, lung, melanoma, and sarcoma [325]. Despite these encouraging results, clinical studies have fallen short, both in terms of efficacy and safety. The most infamous case was a phase II trial in which 17 advanced RCC patients received daily recombinant IL-12 (rhIL-12) for a planned 5 consecutive days every 3 weeks [326]. However, despite reassuring safety data from phase I trials, 12 of the 17 patients required hospitalization due to severe side effects with two treatment-related deaths documented. No patients continued onto cycle 2 due to early termination of the trial. Reported grade 3/4 TRAEs included stomatitis, cytopenias, elevated liver enzymes, and gastrointestinal hemorrhage. Early phase I trials demonstrated that part of the limited efficacy seen in clinical trials relates back to negative feedback signaling through increasing IL-10 level with repeated doses of rhIL-12 [327]. Even when used in combined modality studies, it appeared that IL-12 provided little to no added clinical benefit while simultaneously worsening treatment tolerance [328].

Antibody-IL-12 fusion proteins have also been explored (M9241 also known as NHS-IL12) and although phase I/II trials remain active (NCT04633252, NCT04235777, NCT04756505, and NCT04708470), others have been terminated or suspended in part due to limited clinical efficacy (NCT02994953, NCT04327986). Based on the above issues, research has shifted toward remodeling IL-12 into an adjunct localized therapy as opposed to a non-specific systemic option. Gene therapy with IL-12 soon emerged, initially involving GEN-1, a human plasmid IL-12 (phIL-12) and a DNA delivery system within a lipopolymer which allows for local injections of IL-12-encoding DNA [328]. GEN-1 was first investigated as an intraperitoneal injection and gained traction after a phase I study demonstrated that platinum-sensitive ovarian cancer patients derived an ORR of 50.0% (6/12) with a DCR of 91.7% (11/12) and good tolerability [329]. However, multiple phase I and II trials shortly followed involving platinum-resistant cohorts which failed to replicate these results [330]. Given the above, the focus has since shifted to applying GEN-1 as an adjunct to neoadjuvant chemotherapy for curative intent in ovarian cancer patients, as seen in the phase I OVATION-I trial which found an ORR of 85.7% (12/14) with CR in 14.3% (2/14) of patients and subsequent surgical resection of all macroscopic disease (R0) achieved in 64.3% (9/13) of participants [331]. Translational data also showed reductions in Foxp3, IDO1, PD-1, and PD-L1 in 67–83% of post-treatment biopsies. A randomized phase I/II trial (OVATION-2 and NCT03393884) is currently ongoing to help confirm these encouraging results.

Alternative delivery systems were discussed by H. Kim Lyerly (Duke) at the 2021 China Cancer Immunotherapy workshop include viral (adenovirus and HSV-1) and cellular (CAR-T) vessels, but of the clinical data available regarding viral delivery, little efficacy has been seen [332]. Newer gene therapy agents also include intratumoral IL-12 mRNA (MEDI1191 and SAR441000). In preclinical models, these agents promote Th1 transformation and CD8^+^ T-cell-mediated tumor regression, leading to their advancement into early-phase clinical trials in combination with checkpoint inhibitors for the treatment of advanced solid tumors (NCT03946800 and NCT03871348) [333].

#### TNFα

TNFα is primarily a pro-inflammatory cytokine that assists in the extravasation and activation of effector cells while also triggering apoptosis in aberrant cells [334]. This occurs through the activation of TNF receptor TNFR1, though alternative receptors exist, including TNFR2. TNFR1 is ubiquitously expressed whereas TNFR2 is primarily limited to the CNS, endothelium, and on regulatory immune cells [335]. On abnormal cells, TNFR1 recruits the adaptor proteins TNFR1-associated death domain (TRADD) and Fas-associated death domain (FADD), leading to downstream apoptotic signaling through the caspase cascade [336]. On immune and endothelial cells, TNFR1 drives a pro-inflammatory cascade that leads to the recruitment and expansions of effector cells, which themselves produce TNFα, creating a positive feedback loop [337]. Conversely, TNFR2 signaling provides negative feedback through the activation and expansion of both Tregs and MDSCs [338, 339]. Furthermore, TNFR2 is expressed on a variety of tumor cells, with activation leading to proliferative (NF-κB), angiogenic, and anti-apoptotic effects [340]. TNFR2 expression in cancer subtypes has also been linked to a worsened prognosis [341].

Researchers have engineered ways to create selective TNFR2 inhibitors given the conflicting immune functions of TNFR1 and TNFR2. In culture-based ovarian cancer models (OVCAR3), TNFR2 inhibitors generate anti-tumor activity and reduce the presence of tumor-infiltrating Treg populations [342]. In addition, CRC and lung cancer models involving TNFR2 knockout mice demonstrated reduced metastatic potential and a measurable reduction in suppressive MDSC subsets, further linking TNFR2 to MDSC activity [343]. TNFR2 inhibitors include monoclonal antibodies such as APX601, HFB200301, BI-1808, BITR2101, and SIM0235. Clinical data remain absent, but trials involving HFB200301 (NCT05238883) and BI-1808 (NCT NCT04752826) have already begun accruing participants.

In parallel to immunostimulatory cytokines, some cytokines directly mediate immunosuppression by myeloid cells or exert their inhibitory effect on myeloid cells to promote cancer. Their functions are extremely context dependent. Here we call them immunomodulating cytokines for myeloid cells and described below some important examples.

#### IL-1β

Often a central player in tumor invasion and spread, IL-1β is also integral to the creation of an immunosuppressive network involving TAMs, Tregs, and MDSCs. This has been shown in preclinical HNSCC models where disruption of IL-1β production results a reduction in these tolerogenic cell types and an increase in CD8^+^ T cell presence [344]. TAMs often produce high concentrations of IL-1β as a result of their inflammasomes, further driving recruitment and expansion of MDSCs [345].

Ways of inhibiting IL-1β signaling include the use of recombinant IL-1R antagonists (anakinra), IL-1R accessory protein antagonists (CAN04), IL-1β sequestrants (rilonacept, canakinumab, and gevokizumab), and off-target inhibitors that impact components of either the inflammasome NLRP3 complex or downstream caspase-1 signaling [346, 347].

Oddly enough, interest in IL-1β inhibition as a potential cancer therapy grew considerably following a post hoc analysis from the CANTOS study, a randomized, double-blind, placebo-controlled trial involving the use of canakinumab in 10,061 patients with atherosclerosis and coronary artery disease [348]. Researchers noted that after a median follow-up of 3.7 years, canakinumab recipients had lower rates of cancer mortality (HR 0.49, *p* = 0.0009), lung cancer incidence (HR 0.61, *p* = 0.034), lung cancer mortality (HR 0.23, *p* = 0.0002) compared to those on placebo, along with reductions in C-reactive protein (CRP) and IL-6 levels. However, all-cause mortality was similar between treatment arms (HR 0.94, *p* = 0.31), a finding likely due to sepsis given higher rates of fatal infections seen in the canakinumab arms compared to placebo. This led Novartis to launch four separate large-scale trials (CANOPY-A, CANOPY-N, CANOPY-1, and CANOPY-2) which assessed canakinumab efficacy in the neoadjuvant, adjuvant, and metastatic setting for NSCLC. CANOPY 1 and 2 both failed to meet their primary endpoints of PFS and OS when adding canakinumab to the treatment of metastatic NSCLC, though prespecified subgroup analyses from CANOPY-1 currently suggest that clinically meaningful improvements to PFS and OS were seen in those with increased inflammatory biomarkers [349, 350]. However, in CANOPY-2, rates of fatal infection were elevated with canakinumab therapy (6.7% vs. 1.8%) as previously noted in the CANTOS analysis. The remaining phase II neoadjuvant trial (CANOPY-N, NCT03968419) and phase III adjuvant trial (CANOPY-A, NCT03447769) are still active at this time.

#### IL-6

As mentioned earlier, myeloid precursors are recruited to the bone marrow through cytokines like IL-6. Once differentiated into TAMs, these M2-like cells release IL-6 along with effector cells and tumor cells, thereby promoting tumor plasticity, a term referring to the ability of epithelial cells to transition to mesenchymal phenotypes [351]. This transition has been linked to the aggressiveness and metastatic potential of various cell lines and can also contribute to treatment resistance. One way to combat tumor plasticity while also limiting myeloid recruitment and M2 differentiation is using IL-6 inhibitors. These include IL-6R inhibitors (tocilizumab and sarilumab) and IL-6 sequestrants (siltuximab, sirukumab, olokizumab, and clazakizumab) [352]. The most recognized inhibitor is tocilizumab, a humanized antibody that has received numerous FDA approvals since 2010 in the management of rheumatologic disorders ranging from rheumatoid arthritis to Castleman’s disease. Additionally, tocilizumab is well known for its use in the management of cytokine release syndrome (CRS) during CAR-T therapy.

The first major study to investigate IL-6 sequestrant use in the realm of cancer management was a phase II randomized trial involving the addition of siltuximab to bortezomib, melphalan, and prednisone (VMP) for newly diagnosed, transplant-ineligible multiple myeloma patients [353]. Investigators found no evidence of meaningful clinical improvement in terms of CR rates (27% siltuximab + VMP vs. 22% VMP), ORR (88% siltuximab + VMP vs. 80% VMP), mPFS (17 vs. 17 months) and 1-year OS (88% vs. 88%). This was followed by a separate randomized phase II trial involving relapsed/refractory multiple myeloma patients who received siltuximab or placebo in addition to bortezomib with similar results reported regarding ORR, CR, PFS, and OS [354].

For IL-6R inhibitors, less is known regarding clinical impact. A phase I trial involving tocilzumab, PEGylated IFNα, carboplatin, and doxorubicin in the treatment of epithelial ovarian cancer revealed promising data with an ORR 52.3% (11/21), CR 14.3% (3/21), and DCR of 81.0% (17/21) [355]. The exploratory analysis also found that high-dose tocilizumab (8 mg/kg) resulted in an increase in M1 macrophage presence and secreted IFNγ levels. Regarding adverse events, unique post-market issues have arisen, including increased rates of pancreatitis and gastric perforation, though these events tend to occur in those with predisposing risk factors [356]. More research is necessary to validate the safety and efficacy of this treatment class.

#### IL-10

MDSCs have been shown in murine cancer models to be the primary source of IL-10 within the tumor microenvironment [357]. IL-10 provides autocrine signaling for MDSCs in order to promote their immunosuppressive phenotype. In addition, IL-10 has been shown to lead to a cascade of immunosuppressive effects within the TME, including increased Treg cell activity and inhibition of IL-12 secretion from Th1 helper cells through activation of STAT3 [358]. However, it also carries anti-tumor properties, as illustrated in murine knockout models and in human IL-10R deficiency analyses which found an increased propensity toward the development of malignancies including colon cancer and B cell lymphomas in the absence of IL-10/IL-10R signaling [359–361].

Given these conflicting roles, drug development has remained limited in this area. Recombinant versions of IL-10 have been developed, most promising being pegilodecakin (AM001), a PEGylated human IL-10 with a prolonged half-life. Preclinical data involving pegilodecakin in IL-10^−/−^ mice with chemically induced skin cancers showed that a single dose of systemic pegilodecakin led to increased IFNγ level and cytotoxic T cells, tumor regression, and durable immune memory when mice were rechallenged with tumor cells up to 8 months later [362]. One of the larger clinical trials involving pegilodecakin was the SEQUOIA trial, a phase III study comparing FOLFOX ± pegilodecakin in gemcitabine-refractory pancreatic adenocarcinoma patients [363]. After a median follow-up of 15 months, no difference in median OS (5.8 vs. 6.3 months, *p* = 0.66), median PFS (2.1 vs. 2.1 months, *p* = 0.81), or ORR (4.6% [13/283] vs. 5.6% [16/268]) were reported between the experimental arm and control arm, respectively. Exploratory analyses did show an increase in Granzyme B, IFNγ, and IL-18 levels from baseline, which was more appreciable in the experimental arm, but overall the results were quite underwhelming.

#### TGFβ

TGFβ plays a crucial role in tumor proliferation while also providing immunosuppressive effects on surrounding immune cells, including promotion of M2 phenotypes for TAMs and preventing DC antigen presentation through downregulation of MHC-II [364]. Additionally, TGFβ released from MDSCs can inhibit NK cell activity through interference of IFNγ production while also promoting Treg cell recruitment and expansion. Through the years, multiple TGFβ and TGFβ receptor (TGFβR) inhibitors have emerged to help diminish signaling, including small-molecule TGFβR inhibitors (galunisertib, vactosertib, BMS-986260, LY3200882, LY2157299, PF-06952229, A83-01, SB-431542, RepSox, SM16, and AVID200), TGFβ sequestering monoclonal antibodies (ABBV-151, fresolimumab, SAR439459, NIS793), TGFβR monoclonal antibodies (XPA-42–089), bispecific TGFβR antibodies (BCA101: EGFR; Binstrafusp alfa: PD-L1; *a*-CTLA4-TGFβRII*ecd*: CTLA-4), and TGFβ antisense targeting agents (trabedersen, ISTH0036, TASO-001, gemogenovatucel-T, belagenpumatucel-L) [365, 366].

So far for antisense vaccine approaches, successful preclinical models have failed to translate into promising clinical trial data [367–369]. Part of the reason for this may tie into the complex nature of TGFβ signaling as it has been known to promote anti-tumor signaling in early malignancy settings before later evolving into pro-tumor signals as tumors progress. Additionally, inhibition of TGFβ may lead to compensatory immunosuppressive signaling, preventing tumor regression. Bispecific antibodies such as bintrafusp alfa may help counter this. This past year, pooled data from phase I (NCT02517398) and phase II (NCT03427411) trials involving binstrafusp alfa in advanced, pretreated, checkpoint-naive HPV-associated malignancies were presented at the European Society of Medical Oncology (ESMO) 2021 conference [370]. With a total of 75 patients, the reported ORR was 30.5% (23/75) with a CR rate of 6.7% and a median duration of response was 17.3 months. So similar to immune checkpoint inhibitors alone, a durable response was noted, but the rate of objective responses surpasses that of historical rates seen with PD-1 monotherapy, which is an encouraging sign.

### Strategies to directly target myeloid cells

#### Vaccines

Cancer vaccines were first developed as a way to utilize the body’s immune system to combat malignancies. Initial formulations involved intratumoral whole cell vaccines with limited success due to antigen tolerance; therefore, adjuncts were often required to promote immune response, such as IL-2, GM-CSF, ODNs, and detoxified LPS [371]. Genetic engineering can be used to achieve this as well, as seen with talimogene laherparepvec (T-VEC), an FDA-approved therapy used in the management of stage 3 unresectable melanoma [372]. T-VEC is an oncolytic HSV-1 virus that has been modified with restricted replication in tumor cells and transgene GM-CSF expression [373]. This engineered virotherapy has also been applied to autologous tumor vaccines including GVAX (GM-CSF), FVAX (FLT3L), and TEGVAX (GM-CSF, TLR4 agonist, and TLR7/8 agonist) [374].

Specific targeting was later made possible due to the discovery of tumor-associated antigens (TAAs). These are categorized as either unique antigens (β-catenin-m, HSP70-2/m, Myosin/m, etc.) or shared antigens. Shared antigens include those overexpressed by cancerous cells (HER2, p53, survivn, and livin), antigens differentially expressed by certain tissue types (CEA, PSA, Mammoglobin-A, Tyrosinase, Gp100, MART-1, Melan-A globo-H, Muc1, sTn, and GM2), and antigens unique to germ cells (MAGE, NY-ESO-1, SSX, BAGE, and GAGE) [375]. Despite the discovery of these various antigens, eliciting an immune response to specific vaccines remained difficult without the process of immunostimulatory agents including TLR agonists, so combination therapies are favored. FDA-approved TLR agonists currently used in conjunction with viral vaccination therapies include monophosphoryl lipid A (TLR4 agonist) with hepatitis B and human papilloma virus (HPV), Imiquimod (TLR7 agonist) for anogenital HPV strains, and both Flagellin (TLR5 agonist) and CpG (TLR9 agonist) derivatives for influenza vaccines. While cancer vaccine studies have attempted to extrapolate these immunostimulants into clinical studies, TLR3 agonists have also been repurposed and remain as a leading area of investigation. These agonists typically involve double-stranded RNA complexes like poly-IC or synthetic derivatives for improved stability (rintatolimod) and reduced toxicity (poly-ICLC) which provide a robust innate and adaptive response [376–378]. Data from a phase I trial examining the efficacy and safety of an ovarian cancer peptide vaccine found that NY-ESO-1-specific antibody and CD8^+^ T cell presence improved from 46% (6/13) and 62% (8/13) of participants with vaccine alone to 91% (10/11) and 91% (10/11) when poly-ICLC was added [378, 379].

Another way to improve antigen immunogenicity is by saturating autologous DCs in antigen, whether it be in vivo or ex vivo, in hopes they will then present this antigen to adaptive immune cells. Peripheral blood monocytes can be used to derive dendritic cells ex vivo and antigen loading is possible through use of TAAs or whole tumor cells [380]. A classic example includes sipuleucel-T which is a cellular therapy derived from autologous peripheral monocytes which are activated ex vivo using a recombinant fusion protein (PA2024) consisting of PSA, prostatic acid phosphatase, and GM-CSF [381]. Sipuleucel-T was found to provide a survival benefit across 3 separate double-blind, placebo-controlled, multicenter trials in patients with metastatic castrate-resistant prostate cancer and is now FDA-approved [367, 369]. The majority of DC cancer vaccines that have followed have been lackluster in phase II/III trials, but recent preclinical and phase I data surrounding the identification and use of personalized neoantigens during ex vivo dendritic cell loading appear promising [382–385].

Additional dendritic options include the use of DC-derived exosomes (DCexos) which are inert vesicles expressing MHC-I and II that are unphased by the immunosuppressive state of the tumor microenvironment and provide improved stability and bioavailability [386]. Murine models have shown them to be a viable option for tumor eradication with T cell immunity; however, three early-phase clinical trials involving peptide-loaded DCexos extracted from autologous peripheral monocytes failed to demonstrate meaningful antigen-specific T-cell responses, though NK effector functions were reported [387–390]. The use of protein-loaded DCexos, however, has been documented to induce antigen-specific cytotoxic T cell responses in murine models [391, 392].

#### CAR-M

CAR-T therapy has revolutionized the way clinicians care for patients with B cell malignancies through targeting of CD19 or B cell maturation antigen (BCMA) [393]. However, unique challenges arise when this therapy is applied to solid tumors, including issues surrounding localization, persistence, exhaustion, tumor heterogeneity, and balancing toxicities [394]. Similar to vaccines, these effects are in part due to the presence of MDSCs, so a variety of chimeric co-receptors have been engineered to target key myeloid pathways including CD24 (ONC-782), TR2 (CAR.MUC1/TR2.41BB), and FLT3L among others [395, 396].

Given that pathologic recruitment of monocytes occurs within tumor microenvironments in order to create TAMs, researchers also began work on developing genetically engineered CAR macrophages (CAR-Ms) based on CD19 CAR-T models [397]. Early hypothesis testing involved transducing a human monocytic cell line THP-1 from acute myeloid leukemia with a first generation CD19 CAR-T encoding the intracellular domain of CD3ζ in order to signal antibody-dependent phagocytosis given its structural similarity to FcεRIγ [398]. These CAR-M macrophages successfully engulfed tumor cells in an antigen-specific fashion. Researchers were able to test out further CAR-M iterations by using an adenoviral vector (Ad5F35) to transduce macrophages with CARs targeting solid tumor antigens including mesothelin and HER2, which led to similar success. This was followed by two in vivo ovarian cancer (SKOV3) murine models in which a single infusion of CAR-M therapy led to a significant shrinkage in tumor burden and a considerable prolongation of overall survival, though progressions did eventually occur. Additionally in vivo studies involving biofluorescence also noted CAR-M trafficking and persistence in tumor tissue along with the liver, spleen, and lungs in explanted samples taken 5 days following a single infusion. Overall, CAR-M appears to provide a window of therapeutic opportunity that is currently still in its infancy but will hopefully evolve in years to come. Currently, clinical data are absent with one phase I trial (NCT03608618) involving mesothelin-targeting CAR-M therapy (MCY-M11) currently terminated reportedly as a result of sponsor interests and another phase I trial (NCT04660929) currently underway involving anti-HER2 CAR-M therapy (CT-0508) in HER2-expressing malignancies. In the anti-HER2 trial, one group will receive 3 separate IV infusions over a 5-day period to deliver 5 billion CAR-M cells, whereas another group will receive the 5 billion cells over a single infusion to assess safety, with assessments continuing over a 14-month follow-up period.

Already, separate research has led to the emergence of a new family of CAR-Ms termed CAR-iMacs, which are CAR-M therapies derived from induced pluripotent stem cells (iPSCs) transduced using lentiviral CAR delivery [399]. This process of using iPSCs allows manufacturers to yield high amounts of CAR-M cells from a single collected specimen, further easing production logistics. These CAR-iMacs have already been studied in both in vitro and in vivo solid tumor models with researchers noting antibody-dependent cell phagocytosis in vitro along with in vivo CAR-iMac expansion lasting 2–3 days, tumor burden shrinkage, and CAR-iMac persistence lasting 20–30 days. No word from developers regarding plans for CAR-iMac clinical trials in the near future as further adjustments to improve efficacy and persistence are currently planned.

Finally, non-viral CAR delivery techniques have been discovered, allowing researchers to create in vivo CAR-Ms through the use of available TAMs [400]. Given that macrophages highly overexpress mannose receptors, nanocomplexes such as mannose-conjugated polyethylenimine (MPEI) have been used to target TAMs within the TME and deliver DNA plasmids containing CARs along with IFN-γ in order to polarize them. Preclinical in vivo data involving anti-ALK (anaplastic lymphoma kinase) CAR for the targeting of neuroblastoma models appear to be promising in terms of immunomodulation and tumor shrinkage. If further validated, this new strategy could provide clinicians an off-the-shelf, readily available product that avoids the need for costly ex vivo manufacturing.

## Emerging targets for therapeutic manipulation of myeloid cells

### Siglec-15

Siglec-15 is a sialic acid recognition protein primarily expressed by select myeloid populations that has previously been linked to osteoclast differentiation and bone remodeling, making it a potential target for the management of osteoporosis [401, 402]. However, given its role in macrophage differentiation, it has recently been investigated as a potential tool for activating dormant myeloid cells.

What makes Siglec-15 unique is that unlike other Siglec family members like Siglec-10 which perform intracellular signaling through SHP-1/2 to initiate immunosuppressive actions, Siglec-15 utilizes the adapter protein DAP12 along with a tyrosine kinase called SYK in order to achieve this [403]. Therefore, a unique signaling pathway is available for targeting TAM polarization that avoids redundant antagonism. Siglec-15 ligands include sialic acid-containing glycans such as those with a sialyl-Tn (sTn) structure, a common ligand that is highly associated with a variety of malignancies [404, 405]. When activated in vitro and in vivo using murine models, Siglec-15 has been shown to suppress both T cell proliferation and activation [406]. Furthermore, inhibition of Siglec-15 gives rise to elevated IL-2 and TNFα levels, therefore promoting an inflammatory TME. Mouse models have demonstrated that dual inhibition of Siglec-15 and PD-1 lead to improved tumor responses, including CR, compared to monotherapy alone in either arm [406]. When reviewing expression data from The Cancer Genome Atlas (TCGA), Siglec-15 expression has been shown to be upregulated in a wide variety of malignancies, including CRC, thyroid, endometrial, lung, hepatic, renal, and bladder cancers [406].

Given these findings above, Siglec-15 inhibitors are under development. Currently, NC318, a humanized monoclonal antibody, remains as the sole inhibitor under clinical investigation. A phase I/II trial of NC318 therapy for advanced solid tumors noted no DLTs across dose levels during phase I studies [407]. Adverse events reported included diarrhea (16%), elevated pancreatic enzymes (6–8%), pruritis (6%), and immune-related adverse events that included vitiligo, uveitis, and pneumonitis. For phase II efficacy results, NC318 monotherapy provided an ORR of 4.8% (4/83) with a CR of 1.2% (1/83) and disease control rate (DCR) of 38% (32/83) regardless of PD-L1 or Siglec-15 expression status [408]. Median response was not reported, but 2 patients (CR and PR) remained on therapy beyond 2 years without signs of progression. With NC318 therapy came a dose-dependent increase in soluble Siglec-15, making it a helpful marker for monitoring NC318 activity. Researchers also performed a post hoc analysis using a Siglec-15 immunohistochemistry assay and found that based on screening biopsy samples, Siglec-15 expression on cancer cell membranes was predictive of PFS and duration on therapy [408]. Given the majority of responders to Siglec-15 monotherapy had NSCLC, a phase II trial (NCT04699123) is currently underway comparing NC318 with or without pembrolizumab in patients with advanced NSCLC.

### TREM2

Similar to Siglec-15, TREM2 plays an important role in osteoclast differentiation [409, 410]. Furthermore, both use the adaptor protein DAP12 to transmit intracellular signaling via activation of the tyrosine kinase, Syk [410]. TREM2 expression is restricted within the majority of normal tissue, whereas approximately 75% of cancer types have been shown to express TREM2, making it a suitable target given its wide therapeutic window [411]. Preclinical experiments involving TREM2^+^ DCs and macrophages derived from bone marrow and lung cancer-bearing mice have shown that these innate immune cells inhibit T cell proliferation, secrete higher levels of IL-10, secrete lower levels of IL-12, and phagocytose OVA at reduced capacity [412]. Additionally, the injection of TREM2^+^ DCs into these cancer-bearing mice led to accelerated tumor progression and worsened survival. Individually, the use of IL-10 sequestrants, Syk inhibitors, and TREM-2 antagonists have been demonstrated to independently reverse these effects to varying degrees. Finally, the level of TREM2 presence within TAMs has been positively correlated with tumor staging, including the degree of nodal metastases. Similar inverse trends of TREM2 expression and overall survival have been documented with gastric, hepatic, colorectal, ovarian and breast cancers [411, 413, 414]. Other studies utilizing TREM2^−/−^ mice and TREM2 inhibitors for sarcoma, colorectal, and breast cancer models have demonstrated that TREM2 deficiency leads to improved antigen presentation from TREM2^−/−^ macrophages compared to wild type, along with improved CD8^+^ TIL presence and PD-1 expression, suggesting that TREM2 inhibition may be synergistic with ICI therapy [411].

Based on these findings, development of novel TREM2-targeting agents has been underway. PY314, a humanized monoclonal antibody against TREM2, was among the first to reach clinical trials, with a phase I study (NCT04691375) currently underway comparing PY314 therapy with or without pembrolizumab in patients with advanced solid tumors. While clinical data are currently ongoing, preclinical studies involving PY314 have demonstrated that this anti-TREM2 therapy can provide anti-tumor activity in certain breast cancer models (EMT6) while improving the immune landscape of the TME through increasing the presence of CD8^+^ TILs, NK cells, and MHC-II-expressing TAMs [413]. When combined with anti-PD-L1, PY314 further amplifies these immune cell changes, as seen via flow cytometry and IHC staining. Altogether, TREM2 pathway targeting provides researchers another potential tool in the management of MDSCs.

### MARCO

MARCO represents a pattern recognition scavenger receptor who is expressed constitutively on M2-like subsets of macrophages and whose role has initially been linked to anti-inflammatory changes through cholesterol sequestration in the setting of cardiovascular disease [415]. Analysis of TCGA data shows that MARCO expression is most notable in malignancies of the pancreas, skin, cervix, testicles, thyroid, kidneys, and central nervous system (CNS) [416]. Other cancer types either showed similar or decreased MARCO expression compared to controls. Despite this, MARCO expression has not only been linked to worsened prognosis in glioblastoma and pancreatic cancer, but also gastroesophageal and lung malignancies [417–419]. Moreover, several preclinical models including melanoma, breast and CRC have demonstrated that anti-MARCO monoclonal antibodies not only reduce tumor volumes, but also appear to convert MARCO-expressing TAMs from an M2 to an M1 phenotype while also reducing Treg levels [420]. Separate models (melanoma) have found that the injection of anti-MARCO antibodies into tumor-bearing mice leads to an influx of NK cells and CD8^+^ T cells, and this effect is augmented by the addition of anti-PD-L1 antibodies [421]. Finally, MARCO expression in TAMs is positively correlated with phosphorylation levels of Syk and PI3K [422].

All things considered, MARCO appears to be a promising new immunotherapy target. Given MARCO ligands primarily include acetylated-LDL and negatively charged molecules, sulfatides (negatively charged glycolipids) became one of the first low molecular weight inhibitors to spark interest [423]. While the development of inhibitors against class A scavenger receptors like MARCO remains in its infancy, inhibitors against other scavenger receptor classes such as SR-B1, SR-B2, and LOX-1 are also available for evaluation [424].

### LILRB2

A member of the leukocyte immunoglobulin-like receptor (LILR) family, LILRB2 is found primarily on myeloid immune cells and plays an integral role in providing negative feedback during inflammatory responses through binding to MHC-1 and HLA-G, a non-classical class I molecule [425]. LILRB2 has also been found on hematopoietic stem cells and binds to angiopoietin-like protein 2 (ANGPTL2), leading to the activation of the SHP2 signaling pathway and subsequent cell proliferation [426]. Within malignancies, enrichment of LILRB2 is often noted, including in AML, chronic lymphocytic leukemia (CLL), esophageal cancer, pancreatic cancer, NSCLC, and lobular breast cancers [427]. In vitro, LILRB2 inhibition involving NSCLC cancer cell line A549 leads to significant decrease in cancer migration and proliferation potential [428]. This has been replicated in pancreatic cancer models where silencing ANGPTL2 expression reduced migratory potential through reversion of tumor plasticity [429].

Development of LILRB2 inhibitors is ongoing, including MK-4830, a fully human IgG4 monoclonal antibody specific to LILRB2. Recently published phase I data involving MK-4830 with or without pembrolizumab in advanced solid tumors (NCT03564691) revealed that no DLTs were observed in either arm, with the most common side effects including fatigue (40%), nausea (28%), decreased appetite (22%), and diarrhea (20%) [430]. Regarding efficacy, MK-4830 monotherapy provided an ORR of 2.0% (1/50) and DCR of 24.0% (22/50), whereas combination therapy with pembrolizumab (not including cross overs) led to an ORR of 23.5% (8/34), CR of 2.9% (1/34) and DCR of 50.0% (17/34). At 6 months, DCR was 12% (6/50) and 41.2% (14/34), respectively. Improvements in both cytotoxic T cell levels and PD-L1 positivity were noted in responders, though two responders had a combined positive score (CPS) of 0 following combination therapy. Follow-up trials are in progress, including investigations of MK-4830 in combination with immunotherapy for the treatment of small cell lung cancer (SCC), NSCLC, RCC, CRC, and melanoma. Other LILRB2 inhibitors have also entered early-phase clinical trials, including monoclonal antibodies IO-108 (NCT05054348) and JTX 8064 (NCT04669899).

### CLEVER-1

CLEVER-1 or stabilin 1 has been historically linked to cancer proliferation and spread as noted in *Stab1* knockout mice [431, 432]. However, recent studies also highlight its immunologic impact in TAM polarization, with CLEVER-1 inhibition resulting in TAM conversion to an M1-like phenotype and subsequent T cell activation [433]. Additionally, high concentrations of CLEVER-1 expressing TAMs have been linked to worsened survival outcomes [434]. Given this newer discovery, clinical data in this area have been limited to date. The leading antagonist is bexmarilimab (FP-1305), a humanized IgG4 monoclonal antibody created from Chinese hamster ovary cells that is currently in phase I/II clinical trials involving solid tumors. Preliminary results released at ESMO 2021 suggest good tolerability (most common were fatigue [31%], abdominal pain [23%] and anemia [21%]) but underwhelming response rates with a DCR of 17.2% (19/110) and an ORR of 0% [435].

## Conclusion

The majority of malignant tumors respond poorly to modern ICIs and other immunotherapy agents. Some tumors have innate resistance to immunotherapy, while others acquire resistance over time with many resistance mechanisms traced back to the TME and the lack of myeloid cells. With the advancement of single-cell multi-omics approaches, there is an increased appreciation of the heterogeneity and complexity of myeloid cell composition as described above. However, with a greater understanding of the various myeloid components and the impact of these myeloid subgroups on the TME as well as the tumor response to various therapies, there is a greater potential for manipulation of the myeloid compartment. It is our opinion that combination regimens are most likely to have the greatest impact as illustrated above with the various pleiotropic immune cells and immunocytokines. Employing several agents which target different components of the myeloid component in combination with ICIs or cytotoxic chemotherapy is most likely to have the greatest impact, although with the addition of more therapeutic agents comes the potential for great toxicity.

There has been an eruption of newly developed myeloid-targeted therapies with the majority of these agents still in the clinical trial phase, making it difficult for clinicians to navigate through the available literature and determine which agents appear most promising. In this review, we discussed past and current preclinical and clinical data to provide readers a detailed summary of where each potential target stands and where the future is headed. While T cells have long been the focus of immunotherapy, we believe that the future of immunotherapy will involve targeting myeloid cells.

Key areas of continued research include further investigation into the cross talk among cancer cells, myeloid cells, adaptive immune cells, and surrounding cells like epithelial cells and fibroblasts, to create tolerogenic environments, with the help of single-cell multi-omics technologies. Finally, as our understanding of non-T-cell-based immunotherapy continues to evolve, we are optimistic that the benefit of immunotherapy will be extended to more patients and ultimately save more lives.

## Data Availability

Not applicable.

## Abbreviations

ADCC: Antibody-dependent cellular cytotoxicity
AHR: Aryl hydrocarbon receptor
AML: Acute myeloid leukemia
AMP: Adenosine monophosphate
ANGPTL2: Angiopoietin-like protein 2
APC: Antigen-presenting cell
Arg1: Arginase 1
ATP: Adenosine triphosphate
BCMA: B cell maturation antigen
bFGF: Basic fibroblast growth factor
C/EBPβ: CCAAT/enhancer-binding protein β
CAHON: Chinese American Hematologist and Oncologist Network
CAR-Ms: CAR macrophages
CAR: Chimeric antibody receptor
CCFDIE: China Center for Food and Drug International Exchange
CCL: C–C motif chemokine ligand
cDC: Conventional DC
CDN: Cyclic dinucleotide
CDP: Common dendritic cell precursors
cGAS: Cyclic GMP-AMP synthase
CITE-seq: Cellular indexing of transcriptomes and epitopes by sequencing
CLEVER-1: Common lymphatic endothelial and vascular endothelial receptor 1
CLL: Chronic lymphocytic leukemia
CNS: Central nervous system
COX-2: Cyclooxygenase-2
CpG: Cytosine–phosphate–guanine
CPS: Combined positive score
CR: Complete response
CRC: Colorectal cancer
CRS: Cytokine release syndrome
CSF-1: Colony-stimulating factor 1
CTCL: Cutaneous T cell lymphoma
CTLA-4: Cytotoxic T-lymphocyte-associated protein 4
CXCL: C-X-C motif chemokine ligand
DAMP: Danger-associated molecular patterns
DC: Dendritic cell
DCexos: DC-derived exosomes
DCR: Disease control rate
DNMT: DNA methyltransferase
DOR: Duration of response
dSLIMs: Double stem-loop immunomodulators
ECM: Extracellular matrix
ER: Endoplasmic reticulum
EV: Extracellular vesicle
FADD: Fas-associated death domain
FLT3L: FMS-like tyrosine kinase 3 receptor ligand
GMP: Granulocyte–monocyte progenitor
HATs: Histone acetyltransferases
HCC: Hepatocellular carcinoma
HDAC: Histone deacetylase
HDN: High-density neutrophils
HGF: Hepatocyte growth factor
HMGB1: High-mobility group box 1
HPV: Human papilloma virus
HSC: Hematopoietic stem cell
ICI: Immune checkpoint inhibitor
IDO1: Indoleamine 2,3-dioxygenase 1
IFN: Interferon
iPSC: Induced pluripotent stem cell
ISG: Interferon-stimulated genes
JAKs: Janus kinases
LAP: Leucine–alanine–proline
LDL: Low-density lipoprotein
LDN: Low-density neutrophils
LILR: Leukocyte immunoglobulin-like receptor
LILRB2: Leukocyte immunoglobulin-like receptor B2
LPS: Lipopolysaccharide
M-MDSC: Monocytic MDSC
MARCO: Macrophage receptor with collagenous structure
MDP: Macrophage-dendritic progenitors
MDSCs: Myeloid-derived suppressor cells
MHC: Major histocompatibility complex
MMP: Matrix metallopeptidase
MPEI: Mannose-conjugated polyethyleneimine
mPFS: Median progression-free survival
mregDC: Mature DCs enriched in immunoregulatory molecules
NAD^+^: Nicotinamide adenine dinucleotide
NADPH: Nicotinamide adenine dinucleotide phosphate
NE: Neutrophil elastase
NETs: Neutrophil extracellular traps
NHL: Non-Hodgkin lymphoma
NLR: Neutrophil-to-lymphocyte ratio
NMPA: China National Medical Product Administration
NSCLC: Non-small cell lung cancer
ODN: Oligodeoxynucleotide
ORR: Objective response rate
PAMP: Pathogen-associated molecular patterns
PD-1: Programmed cell death protein 1
PD-L1: Programmed death-ligand 1
pDC: Plasmacytoid DC
PDGF: Platelet-derived growth factor
PI3Kγ: Phosphatidylinositol 3-kinase gamma
PKCβII: Protein kinase C βII
PMN-MDSC: Granulocytic/polymorphonuclear MDSC
PNT: Peroxynitrite
PVNS: Pigmented villonodular synovitis
RCC: Renal cell carcinoma
rhuFLT3L: Recombinant human FLT3 ligand
rIFNα2: Recombinant IFNα2
RNS: Reactive nitrogen species
ROS: Reactive oxygen species
SCC: Small cell lung cancer
SHP-1/2: Src homology domain-containing tyrosine phosphatases 1/2
Siglec-10: Sialic acid-binding immunoglobulin-type lectin-10
STAT3: Signal transducer and activator of transcription
STING: Stimulator of interferon genes
TAAs: Tumor-associated antigens
TAM: Tumor-associated macrophage
TAN: Tumor-associated neutrophils
TCGA: The Cancer Genome Atlas
TCR: T cell receptor
TDEs: Tumor-derived exosomes
TDO: Tryptophan-2,3-dioxygenase
TGCT: Tenosynovial giant cell tumor
TGFβ: Transforming growth factor β
TIL: Tumor-infiltrating lymphocytes
TLR: Toll-like receptors
TME: Tumor microenvironment
TNBC: Triple-negative breast cancer
TNFα: Tumor necrosis factor α
TRAAC: Toll-like receptor agonist antibody conjugates
TRADD: TNFR1-associated death domain
TRAEs: Treatment-related adverse events
Treg: Regulatory T cell
TREM2: Triggering receptor expressed on myeloid cells 2
TRM: Tissue-resident macrophage
TRPM2: Transient receptor potential melastatin 2
T-VEC: Talimogene laherparepvec
VEGF: Vascular endothelial growth factor
VISTA: V-domain immunoglobulin suppressor of T cell activation
